# Hypothalamic leptin action is mediated by histone deacetylase 5

**DOI:** 10.1038/ncomms10782

**Published:** 2016-02-29

**Authors:** Dhiraj G. Kabra, Katrin Pfuhlmann, Cristina García-Cáceres, Sonja C. Schriever, Veronica Casquero García, Adam Fiseha Kebede, Esther Fuente-Martin, Chitrang Trivedi, Kristy Heppner, N. Henriette Uhlenhaut, Beata Legutko, Uma D. Kabra, Yuanqing Gao, Chun-Xia Yi, Carmelo Quarta, Christoffer Clemmensen, Brian Finan, Timo D. Müller, Carola W. Meyer, Marcelo Paez-Pereda, Kerstin Stemmer, Stephen C. Woods, Diego Perez-Tilve, Robert Schneider, Eric N. Olson, Matthias H. Tschöp, Paul T. Pfluger

**Affiliations:** 1 Helmholtz Diabetes Center, Helmholtz Zentrum München, 85764 Neuherberg, Germany; 2 Division of Metabolic Diseases, Technische Universität München, 80333 Munich, Germany; 3 Institut de Génétique et de Biologie Moléculaire et Cellulaire, CNRS UMR 7104, INSERM U 964, Université de Strasbourg, Illkirch 67404, France; 4 Department of Pharmacology & Toxicology, Zydus Research Centre, Cadila Healthcare Limited, Sarkhej-Bavla N.H. No. 8A, Moraiya, Ahmedabad 382210, India; 5 Metabolic Diseases Institute, Division of Endocrinology, Department of Internal Medicine, University of Cincinnati, Cincinnati, Ohio 45237, USA; 6 Max Planck Institute of Psychiatry, Kraepelinstr. 2-10, 80804 München, Germany; 7 Department of Molecular Biology, UT Southwestern Medical Center, Dallas, Texas 75390, USA

## Abstract

Hypothalamic leptin signalling has a key role in food intake and energy-balance control and is often impaired in obese individuals. Here we identify histone deacetylase 5 (HDAC5) as a regulator of leptin signalling and organismal energy balance. Global HDAC5 KO mice have increased food intake and greater diet-induced obesity when fed high-fat diet. Pharmacological and genetic inhibition of HDAC5 activity in the mediobasal hypothalamus increases food intake and modulates pathways implicated in leptin signalling. We show HDAC5 directly regulates STAT3 localization and transcriptional activity via reciprocal STAT3 deacetylation at Lys685 and phosphorylation at Tyr705. *In vivo*, leptin sensitivity is substantially impaired in HDAC5 loss-of-function mice. Hypothalamic HDAC5 overexpression improves leptin action and partially protects against HFD-induced leptin resistance and obesity. Overall, our data suggest that hypothalamic HDAC5 activity is a regulator of leptin signalling that adapts food intake and body weight to our dietary environment.

## Abstract (web summary)

Histone deacetylases (HDACs) regulate energy metabolism in peripheral tissues, but whether HDACs expressed in the brain influence systemic metabolism is unknown. Here the authors show that hypothalamic HDAC5 expression is affected by the diet and HDAC5 regulates leptin sensitivity by deacetylating STAT3.

## Graphical abstract

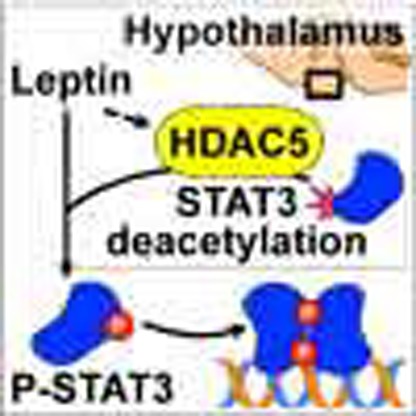

Over the 20 years since leptin's discovery, much has been learned about its downstream targets in the central nervous system and the complex regulatory network controlling food intake, body weight and systemic metabolism^1^,^2^,^3^,^4^,^5^,^6^,^7^. Diverse metabolic and genetic approaches have identified the hypothalamus as the primary location for leptin-responsive signalling cascades responsible for controlling energy homeostasis, particularly acting through the melanocortin system^3^,^8^,^9^,^10^,^11^,^12^,^13^. Centrally projecting neurons such as proopiomelanocortin (POMC) or Agouti-related protein (AGRP) neurons within the hypothalamic arcuate nucleus (ARC) are primary leptin targets and stimulate or inhibit second-order neurons via GABAergic and glutamatergic projections to numerous brain areas and hypothalamic nuclei, respectively^14^,^15^. POMC neurons also secrete several bioactive peptide fragments cleaved from POMC including αMSH, which activates MC receptors 3 and 4 (MC3r/MC4r) to elicit hypophagia^1^,^3^. Agouti-related protein (AGRP) neurons within the ARC secrete AGRP, which directly competes with αMSH as an inverse MC3r/MC4r agonist to promote hyperphagia.

The failure to activate leptin signalling in AGRP- and POMC neurons is a key feature of ‘leptin resistance' and represents a pivotal event in the development of diet-induced obesity and its comorbid sequelae^8^,^9^,^11^,^12^,^13^. Leptin resistance may be in part a heritable trait, but even siblings often display large variations in the propensity to develop leptin resistance despite similar genetics and a comparable socioeconomic environment^16^,^17^,^18^. Exactly how gene-environment interactions predispose an organism to aberrant leptin signalling remains elusive. We here pursued the hypothesis that deleterious post-translational modifications of leptin signalling components occur in response to diets and predispose to obesity.

A key mechanism to modulate gene–environment interactions is the acetylation of lysine residues in histone and non-histone proteins, tightly controlled by histone acetyltransferases (HATs) and histone deacyetylases (HDACs). HDAC class III family members such as SIRT1 or SIRT3 are well known regulators of energy and glucose metabolism^16^. Class IIa family members HDAC 4, 5, 7 and 9 excert partially redundant activity in hepatic glucose control^17^, adipogenesis and adipose tissue (dys)function^19^,^20^,^21^ and muscle differentiation^22^. Class IIa family members were further shown to be expressed in hypothalamic AGRP and POMC neurons^23^,^24^, but a central function in metabolic control and leptin signalling has not been established.

We here aimed to assess whether class IIa family member HDAC5 plays a role in the central control of metabolic homeostasis and leptin signalling. Our specific interest in HDAC5 was triggered by the following observations. HDAC5 expression is relatively restricted, with highest expression observed in muscle, heart and brain^25^,^26^. HDAC5 is expressed in POMC and AGRP neurons and regulated by the dietary environment^23^,^24^, and HDAC5 is shown to be the central epigenetic integrator of an animal's adaptive response to short-term and long-term emotional stress^26^. We here report that HDAC5-mediated deacetylation of STAT3 is an important regulator of hypothalamic leptin signalling and appropriate central nervous system (CNS) control of energy homeostasis.

## Results

### HDAC5 expression is regulated by dietary lipids and leptin

**Figure 1:**
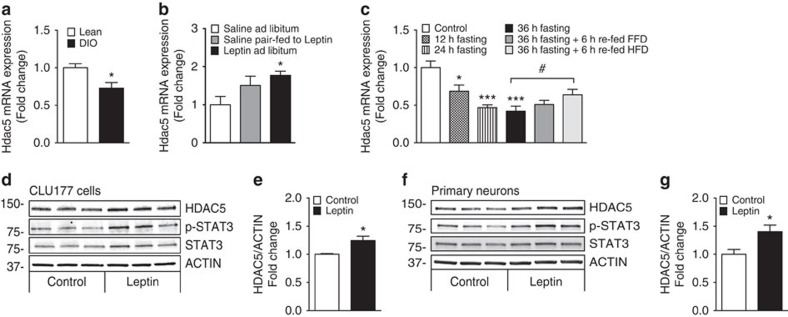
Hypothalamic histone deacetylase 5 (HDAC5) expression is regulated by nutrient availability and leptin sensitivity. Hypothalamic *Hdac5* mRNA levels were assessed by real-time PCR in (**a**) lean versus diet-induced obese (DIO) male mice (*n*=7) and (**b**) male *Lep*^*ob*^ mice treated subcutaneously for 6 days with either saline or 1 mg kg^−1^ leptin (*n*=5–6). Saline-treated mice were fed *ad libitum* (*ad lib*) or pair-fed to the lower food consumption of the Leptin *ad lib* group. (**c**) Hypothalamic *Hdac5* expression was assessed in male C57BL/6J mice that were subjected to 12, 24 or 36 h of fasting as well as refeeding for 6 h with fat-free diet (FFD) or high-fat diet (HFD; *n*=6–8). HDAC5, total STAT3, p-STAT3 and ACTIN immunoblots and densitometric analyses of HDAC5 /ACTIN ratios in CLU177 cells (**d**,**e**) and primary neurons (**f**,**g**) treated for 24 h with leptin. Values are means±s.e.m. Statistical analyses were done by two-tailed unpaired Student's *t*-test (**a**,**e**,**g**) or by One-Way ANOVA followed by Bonferroni (**b**) or Dunnet (**c**) *post hoc* tests. *P<0.05, **P<0.01 and ***P<0.001 versus Control; #*P*<0.05 versus 36-h fasting.

We first aimed to delineate whether the expression of epigenetic regulator HDAC5 is affected by nutrient availability and the metabolic state. Hypothalamic *Hdac5* expression was reduced in high-fat diet-induced obese (DIO) mice when compared with chow-fed lean controls (Fig. 1a). To determine the relevance of HDAC5 for CNS leptin action, we assessed hypothalamic *Hdac5* expression in leptin-deficient (Lep^ob^) mice with and without leptin replacement. Rescue of leptin deficiency normalized hypothalamic *Hdac5* expression, which indicates that the state of leptin sensitivity directly affects hypothalamic *Hdac5* gene expression (Fig. 1b). The concomitant non-significant increase of *Hdac5* expression in saline-treated Lep^ob^ mice that were pair fed to leptin-treated Lep^ob^ mice (Fig. 1b) prompted us to assess a direct impact of dietary nutrients on hypothalamic *Hdac5* gene expression. Mice subjected to prolonged fasting revealed a decrease in *Hdac5* gene expression and an increase after acute refeeding with high-fat diet (HFD; Fig. 1c). Because refeeding with fat-free diet did not elevate hypothalamic *Hdac5* messenger RNA (mRNA) expression, the data suggest that dietary lipids are important for the regulation of HDAC5 in the CNS (Fig. 1c).

HFD-induced obesity did not affect hypothalamic expression levels of HDAC class IIa family members *Hdac4* and *9* (Supplementary Fig. 1a). CNS leptin action but not pair-feeding induced the expression of hypothalamic *Hdac4* but not *Hdac9* (Supplementary Fig. 1b). Hypothalamic *Hdac4* and *9* mRNA levels were increased slightly with short-term food deprivation but decreased after prolonged fasting. Refeeding with fat-free diet and HFD decreased hypothalamic *Hdac4* mRNA levels. In contrast, hypothalamic *Hdac9* levels were slightly increased with refeeding of HFD, and *Hdac7* mRNA levels remained unaffected by changes in nutrient availability (Supplementary Fig. 1c). Overall, *Hdac5* emerged as the most consistent nutrient-responsive member of the HDAC IIa family within the hypothalamus.

We next used the hypothalamic cell line CLU177 and primary hypothalamic neurons to delineate the impact of direct leptin action from nutrient availability on HDAC5 expression. Leptin treatment significantly increased HDAC5 protein levels in CLU177 cells treated with 1 μg ml^−1^ (Fig. 1d,e) and hypothalamic primary neuron with 100 ng ml^−1^ leptin (Fig. 1f,g) for 24 h. The increase in HDAC5 levels was paralleled by a 1.4-fold increase in phosphorylation of the known leptin target STAT3 in CLU177 cells and primary neurons, respectively. In summary, hypothalamic *Hdac5* expression selectively and specifically responds to leptin signalling, to the availability of dietary lipids, and, possibly, body adiposity.

### HDAC5 ablation enhances responsiveness to HFD

**Figure 2:**
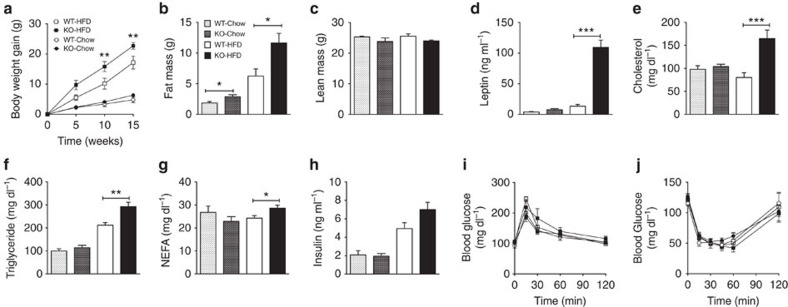
HDAC5 knock-out mice are prone to diet-induced obesity. Male HDAC5 WT and KO littermates were subjected to either chow or HFD and evaluated for (**a**) body weight gain over a total of 15 weeks; (**b**) fat and (**c**) lean mass after 8 weeks of chow or HFD exposure (*n*=6–8). After 10 weeks of HFD and 15 weeks of chow exposure, plasma (**d**) leptin, (**e**) cholesterol, (**f**) triglyceride, (**g**) non-esterified fatty acid and (**h**) insulin levels were measured in additional cohorts of chow- or HFD-fed HDAC5 WT (*n*=6–7) and KO mice (*n*=9–10). (**i**) Glucose tolerance and (**j**) insulin tolerance tests were carried out after 8 weeks of diet exposure (*n*=4–8). Values represent means±s.e.m. Statistical analyses were done by either two-way ANOVA with Bonferroni *post hoc* tests (**a**,**i**,**j**), or two-tailed unpaired Student's *t*-test (**b**–**h**). *P<0.05, **P<0.01 and ***P<0.001.

Both male and female global HDAC5 knock-out (KO) mice displayed greater body weight gain than wild-type (WT) littermates when fed HFD for 15 weeks (Fig. 2a; Supplementary Fig. 2a, body weights at weeks 0 and 15 of HFD exposure: male KO 26.31±1.07 g to 47.78±1.60 g; female KO 22.78±1.03 to 43.62±2.10 g; male WT 29.27±0.90 g to 47.39±2.89 g; and female WT 23.01±0.66 g to 35.58±2.36 g), further suggesting a role of HDAC5 in the CNS response to dietary lipids. There were no body weight differences when the mice consumed chow diet for 15 weeks (Fig. 2a Supplementary Fig. 2a, body weights at weeks 0 and 15 of HFD exposure: male KO mice: 26.87±1.13 g to 33.23±1.24 g; female KO mice: 20.80±0.53 g to 23.25±0.65 g; male WT mice: 28.44±0.49 g to 34.41±1.41 g; female WT mice: 20.88±0.87 g to 24.37±1.20 g). The additional body-weight gain of HDAC5-deficient mice on HFD was a consequence of increased fat mass in both sexes, and was also apparent in male chow-fed HDAC5 KO mice when compared with WT controls (male mice: Fig. 2b; female mice: Supplementary Fig. 2b), likely reflecting the lower, but still considerable, fat content of the standard chow diet. Male HFD-fed HDAC5 KO mice displayed larger epididymal white adipose tissue and combined interscapular fat pads consisting of white and brown adipose tissue (BAT; Supplementary Fig. 2c), as well as increased liver weight (Supplementary Table 1) and liver triglyceride levels (Supplementary Fig. 2d). Nasoanal length (data not shown) and lean mass (male mice: Fig. 2c; female mice: Supplementary Fig. 2e) did not differ between genotypes. Plasma leptin levels were approximately eightfold higher in male HFD-fed HDAC KO and approximately twofold higher in female HFD-fed HDAC KO mice compared with HFD-fed WT controls (male mice: Fig. 2d; female mice: Supplementary Fig. 2f). HDAC5 KO mice consuming HFD also had elevated fasting plasma cholesterol, triglycerides and non-esterified free fatty acids compared with WT littermates, whereas these biochemical parameters did not differ between genotypes fed the chow diet (Fig. 2e–g).

A previous report suggested that virally mediated knockdown of HDAC class IIa family members 4, 5 and 7 in the liver improves systemic glucose homeostasis in mice^17^. We aimed to assess the impact of global HDAC5 ablation on glucose homeostasis in male and female mice exposed to chow or HFD. In both genotypes, HFD exposure but not chow feeding, resulted in increased fasting plasma insulin levels, with a trend towards higher insulin levels in HFD-fed HDAC5 KO mice, compared with HFD-fed WT controls (male mice, *P*=0.054, two-tailed *t*-test; Fig. 2h; female mice, *P*=0.1399, two-tailed *t*-test; Supplementary Fig. 3a). HFD exposure for 8 weeks (male mice: Fig. 2i) or 15 weeks (female mice: Supplementary Fig. 3b; male mice: Supplementary Fig. 3c) incurred comparable glucose intolerance in both WT and HDAC5 KO mice. Insulin tolerance did not differ between male HDAC5 KO mice and WT littermates after 8 weeks chow or HFD feeding (Fig. 2j). The hepatic expression of gluconeogenic genes phosphoenolpyruvate carboxykinase 1 (*Pck1*) and glucose 6-phosphatase (*G6pc*) was similar in WT and HDAC5 KO mice exposed to 10 weeks of HFD (Supplementary Fig. 3d). Notably, HFD-fed HDAC5 KO mice displayed increased glucokinase (*Gck1*) and fibroblast growth factor 21 (*Fgf21*) expression, and enhanced expression of lipogenic enzymes such as fatty acid synthase (*Fasn*) or acetyl-CoA carboxylase 1 and 2 (*Acc1*; *Acc2*) (Supplementary Fig. 3d). Collectively, our data do not support a systemic glucoregulatory role of HDAC5. Rather, our results indicate that HDAC5 has a specific and important role in the molecular processes leading to HFD-induced obesity and associated hyperleptinemia.

### Hyperphagia drives obesity in HDAC5 KO mice

**Figure 3:**
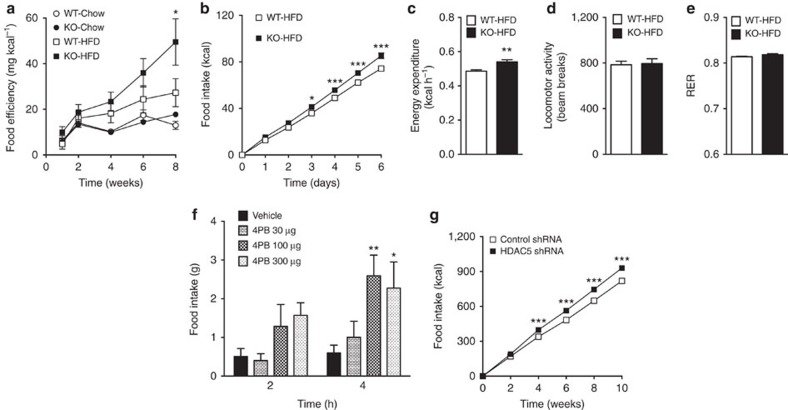
Hypothalamic disruption of HDAC5 activity causes hyperphagia in lean mice and rats. (**a**) Feed efficiency was calculated from male HDAC5 KO compared with WT littermates subjected to chow or HFD for in total 8 weeks (*n*=6–8). Acute effects of HFD exposure on (**b**) daily food intake, (**c**) energy expenditure, (**d**) locomotor activity and (**e**) respiratory exchange ratio (RER) were assessed in male HDAC5 WT and KO littermates for a total of 6 days (*n*=7–8). (**f**) Increased food intake in male rats subjected to a single third ventricular injection of HDAC IIa inhibitor 4-phenylbutyric acid (4PB) compared with vehicle controls (*n*=6–7). (**g**) Weekly food intake over 10 weeks of HFD feeding in male C57BL/6J mice with lentiviral shRNA-mediated knockdown of HDAC5 or non-silencing shRNA in the MBH (*n*=12–13). Values represent means±s.e.m. Statistical analyses were done by either two-way ANOVA and Bonferroni *post hoc* tests (**a**,**b**,**f**,**g**) or two-tailed unpaired Student's *t*-test (**c**–**e**). (**a**) *P<0.05 versus WT-HFD, (**b**–**e**,**g**) *P<0.05, **P<0.01 and ***P<0.001; (**f**) *P<0.05 and **P<0.01 versus vehicle.

Our finding of significantly higher feed efficiency (Fig. 3a) in male HDAC5 KO mice chronically consuming HFD prompted us to delineate whether HDAC5 ablation is causally linked with hyperphagia or dysfunctional energy expenditure. Compared with WT controls, male HDAC5 KO mice acutely exposed to HFD for one week displayed increased food intake (Fig. 3b) and energy expenditure (Fig. 3c; Supplementary Fig. 3e). A correlation of energy expenditure with food intake suggested that HDAC5 KO mice expend more calories to partially compensate for the increase in calorie intake (Supplementary Fig. 3f). The increase in energy expenditure was not due to hyperactivity, as revealed by comparable locomotor activity between the two genotypes (Fig. 3d), or due to BAT activity, as revealed by unchanged BAT weight (Supplementary Table 1) and unchanged gene expression of uncoupling protein 1 (*Ucp1*), *Ucp2*, peroxisome proliferator-activated receptor gamma coactivator 1-alpha (*Ppargc1a)* and cell death-inducing dffa-like effector A (*Cidea*), a BAT-specific regulator of UCP1 activity, thermogenesis and lipolysis^31^ (Supplementary Fig. 3g). Moreover, fuel preference (nutrient partitioning) was similar between genotypes (Fig. 3e). These results suggest that food intake is a causative factor for the obese phenotype and the perturbed compensatory response to consuming HFD of HDAC5 KO mice, implicating the CNS, and particularly the hypothalamus, rather than peripheral sites of action for the role of HDAC5 in the control of energy homeostasis.

### Loss of hypothalamic HDAC5 function results in hyperphagia

Administration of the HDAC class II inhibitor 4-phenyl butyrate (4PB)^32^ into the third cerebral ventricle of rats elicited hyperphagia for several hours (Fig. 3f). Lentiviral delivery of small hairpin RNA (shRNA) to knockdown HDAC5 locally in the mediobasal hypothalamus (MBH), a metabolic control center comprised mostly of the arcuate nucleus and ventromedial hypothalamus, resulted in chronic hyperphagia (Fig. 3g). Collectively, these pharmacological and genetic data demonstrate that hypothalamic HDAC5 is an essential regulator of food intake and suggest that leptin signalling may be involved in this process.

### HDAC5 disruption impairs POMC expression

**Figure 4:**
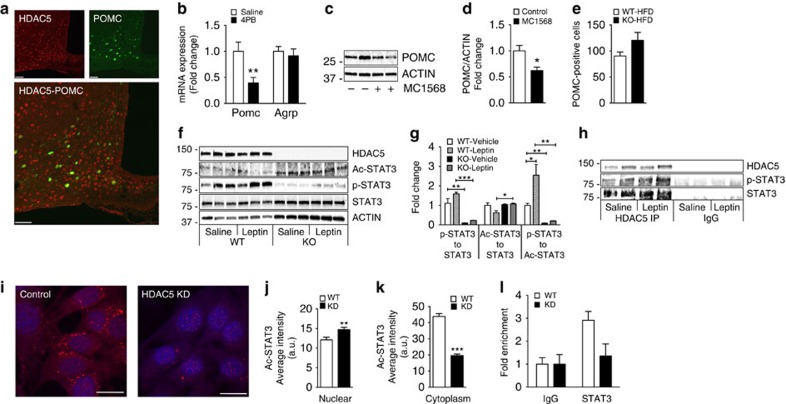
Hypothalamic disruption of HDAC5 activity impairs POMC expression and STAT3 signalling: (**a**) Hypothalamic slices of male POMC–GFP mice were subjected to immunostaining with anti-rabbit HDAC5 antibody, and revealed widespread HDAC5 expression and colocalization to ∼68.62% of all POMC neurons. (*n*=4 slices, Scale bar, 50 μm) (**b**) Hypothalamic mRNA levels of *Pomc* and *Agrp* in rats 2 h after 3rd ventricular injection of 4-phenylbutyric acid (4-PB) or vehicle (*n*=8). (**c**,**d**) Western blot and densitometric analysis of POMC and ACTIN protein levels in CLU177 cells 24 h after treatment with the selective class IIa HDAC inhibitor MC1568 (*n*=3). (**e**) The number of stained cells in hypothalamic slices after immunohistochemical detection of POMC was counted in male HDAC5 WT and KO mice subjected to 10 weeks of HFD feeding (*n*=3). (**f**,**g**) Representative western blot and densitometric analysis of reference protein ACTIN, HDAC5 and total STAT3, Ac-STAT3^K685^ and p-STAT3^T705^ in primary hypothalamic neurons isolated from KO and WT mice that were subjected to saline or leptin (100 ng ml^−1^) treatment for 30 min (*n*=3). (**h**) Western blot for HDAC5 STAT3 and pSTAT3^T705^ following immunoprecipitation with HDAC5 antibody from control CLU177 cells. (**i**) Representative confocal images of Ac-STAT3^K685^ (red colour) in WT and CLU177 cells with stable knockdown of HDAC5. Nuclei are counterstained using DAPI (blue), Scale bar, 15 μm. The cytoplasmic (**j**) versus nuclear (**k**) localization of Ac-STAT3^K685^ was assessed using ImageJ software. (**l**) Chromatin immunoprecipitation (ChIP) with total STAT3 antibody reveals diminished STAT3 recruitment to the POMC promoter in CLU177 cells with HDAC5 KD, compared with WT controls (*n*=3). Values represent means±s.e.m. Statistical analyses were done either by two-tailed unpaired Student's *t*-tests (**b**,**d**,**e**,**j**,**k**,**l**) or one-way ANOVA followed by Bonferroni *post hoc* tests (**g**). *P<0.05 and **P<0.01.

HDAC5 was widely expressed in the arcuate nucleus of mice. HDAC5 expression was found in 68.62% of all POMC–green fluorescent protein (GFP)-carrying neurons (Fig. 4a), and in 83.80% of all NPY–GFP carrying neurons (Supplementary Fig. 4a). No expression was found in non-neuronal astrocytes or microglia (Supplementary Fig. 4b,c). Importantly, acute pharmacological inhibition as well as chronic lentiviral knockdown of hypothalamic HDAC5 in adult rats or mice had no effect on *Agrp* expression but consistently suppressed *Pomc* gene expression (Fig. 4b; Supplementary Fig. 4d). In hypothalamic CLU177 cells, POMC protein levels were decreased by the selective HDAC class IIa inhibitor MC1568 (Fig. 4c,d). Moreover, compared with what occurred in HFD-fed WT littermates, HFD-fed HDAC5 KO mice had decreased *Pomc* mRNA expression in microdissected ARCs (Supplementary Fig. 4e). We confirmed equal numbers of hypothalamic POMC neurons in adult chow fed (Supplementary Fig. 4f) and HFD fed (Fig. 4e) WT and HDAC5 KO mice, which excludes developmental loss of hypothalamic neurons following germ-line ablation of HDAC5 as the explanation for the repressed POMC expression observed.

### HDAC5 controls leptin signalling via STAT3 deacetylation

We next determined whether genetic manipulation of HDAC5 would affect key components of leptin signalling. Loss of HDAC5 altered hypothalamic expression levels of many intermediates of the canonical leptin-signalling pathway, with decreased mRNA levels of *Stat3*, *Pi3k, Ptp1b and Socs3* in chow-fed HDAC5 WT and KO mice (Supplementary Fig. 4g), decreased *LepR*, *Stat3*, *Ptp1b*, *Socs3* and *Pp2a* mRNA levels in HFD-fed HDAC5 WT and KO mice (Supplementary Fig. 4h), and decreased *LepR*, *Stat3* and *Ptp1b* as well as increased protein phosphatase 2 (*Pp2a*) mRNA levels in primary neurons from WT and KO mice (Supplementary Fig. 4i).

Since STAT3 is a direct transcriptional activator of POMC gene expression^27^,^28^,^29^, we assessed STAT3 protein levels, Tyr 705 phosphorylation and Lys 685 acetylation, respectively. Compared with primary neuronal cultures from WT controls, primary neuronal cultures isolated from HDAC5 KO mice had increased Lys685 acetylation (Ac-STAT3) but diminished STAT3 phosphorylation (p-STAT3) at baseline and after leptin stimulation (Fig. 4f), leading to a decreased p-STAT3/STAT3 and p-STAT3/Ac-STAT3 ratio (Fig. 4g). We corroborated diminished STAT3 phosphorylation at Tyr705 and increased STAT3 acetylation at Lys685 in hypothalamic CLU177 cells with shRNA-mediated knockdown of HDAC5 (Supplementary Fig. 4j). Adenoviral overexpression of HDAC5 in CLU177 cells and in hypothalamic primary neurons led to a decrease in STAT3^K685^ acetylation and a concomitant increase in POMC protein expression, respectively (Supplementary Fig. 4k–m). Co-immunoprecipitation experiments confirmed that HDAC5 directly interacts with STAT3, allowing for deacetylation at its Lys685 (Fig. 4h).

CLU177 control cells with stable expression of scrambled shRNA displayed a uniform distribution of Ac-STAT3^K685^ in the cytosol and nucleus; in contrast, stable shRNA-mediated knockdown of HDAC5 induced a redistribution of Ac-STAT3^K685^, which was detected almost exclusively in the nucleus (Fig. 4i–k). Last, by using chromatin immunoprecipitation PCR in CLU177 cells with knockdown of HDAC5, we observed a strong trend towards reduced binding of STAT3 to its *cis*-regulatory element near the transcriptional start site of the *Pomc* promoter (*P*=0.075, two-tailed *t*-test; Fig. 4l). These results collectively identify HDAC5 as an essential component of the functional leptin signalling cascade and suggest a potential mechanistic role in fine-tuning STAT3 deacetylation and phosphorylation and its cellular localization.

### HDAC5 affects leptin action and body weight *in vivo*

**Figure 5:**
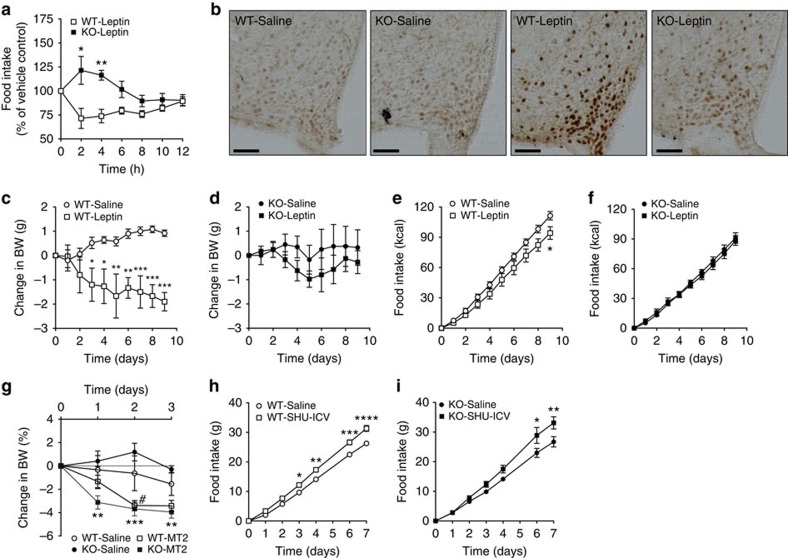
Hypothalamic HDAC5 knockdown impedes leptin action: (**a**) Overnight food intake in chow-fed male HDAC5 WT and KO littermates (*n*=8) subjected to a single intraperitoneal (i.p.) injection of vehicle saline or leptin (5 mg kg^−1^) at the beginning of the dark cycle. (**b**) Representative immunohistochemical detection of STAT3 phosphorylation in hypothalamic slices of chow-fed male HDAC5 KO and WT littermates 30 min after a single i.p. injection of vehicle saline or leptin (5 mg kg^−1^, *n*=3, Scale bar, 50 μm). (**c**,**d**) Body weight and (**e**,**f**) food intake in chow-fed male WT and HDAC5 KO mice subjected to chronic ICV leptin (1 μg/day) or PBS infusion via Alzet minipumps (*n*=3–5). (**g**) Body weight changes of male chow-fed WT and HDAC5 KO mice subjected to daily i.p. injections of melanocortin receptor agonist MT2 (1 mg kg^−1^ , *n*=3–5). (**h**,**i**) Food intake of male chow-fed WT and HDAC5 KO mice (*n*=3–5) after chronic intracerebroventricular infusion of melanocortin receptor antagonist SHU9119 (24 nmol/day). Values represent means±s.e.m. Statistical analyses were done by using two-way ANOVA followed by Bonferroni *post hoc* tests (**a**–**i**). (**a**,**c**,**e**,**h**,**i**) *P<0.05, **P<0.01, ***P<0.001 and ****P<0.0001; (**g**) #*P*<0.05 WT-Saline versus WT-MT2, **P<0.01 and ***P<0.001 KO-Saline versus KO-MT2.

Next, we administered leptin (intraperitoneal (i.p.)) to WT and HDAC5 KO mice and measured food intake and STAT3 activation. Leptin significantly decreased food intake in WT mice but had no effect in KO mice (Fig. 5a), proving that HDAC5 is functionally required for the hypophagic component of leptin action. Consistent with this, leptin-induced STAT3 phosphorylation was markedly blunted in HDAC5 KO mice (Fig. 5b). Intracerebroventricular (i.c.v.) infusion with leptin for 9 days decreased body weight in WT mice (Fig. 5c), but not in HDAC5 KO mice (Fig. 5d). The i.c.v. leptin also decreased food intake in WT (Fig. 5e), but not in HDAC5 KO mice (Fig. 5f).

We further assessed whether blunted leptin action on food intake is a consequence of dysfunctional melanocortin signalling. Daily i.p. treatment of male chow-fed WT and HDAC5 KO mice with melanocortin receptor 3 and 4 (MCR3/MCR4) agonist MT-II induced a comparable decrease in body weight in both chow-fed WT and HDAC5 KO mice (Fig. 5g). Intracerebroventricular infusion of the MCR3/MCR4 antagonist/inverse agonist SHU9119 increased food intake in both genotypes (Fig. 5h,i), confirming that melanocortin signalling at the level or downstream of MC3r/MC4r remains intact in HDAC5 KO mice.

### HDAC5 overexpression improves diet-induced obesity

**Figure 6:**
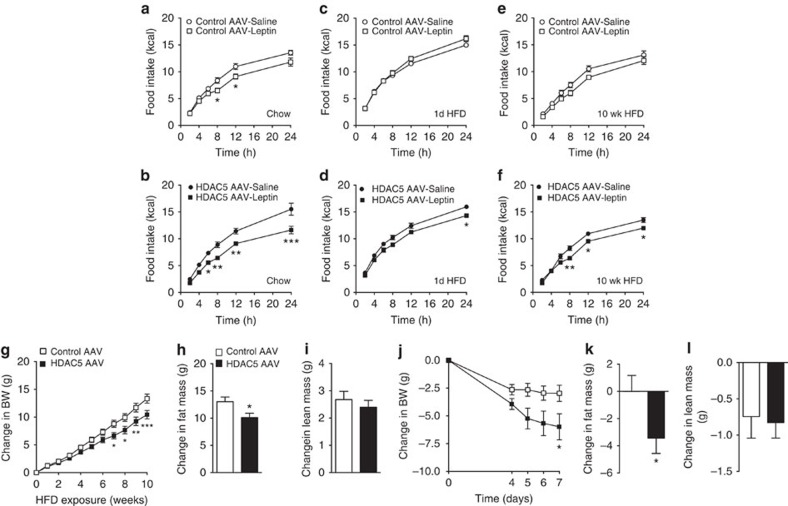
Hypothalamic HDAC5 overexpression improves leptin sensitivity and ameliorates diet-induced obesity: One week after AAV-mediated overexpression of HDAC5 or control GFP in the MBH, chow-fed male C57BL/6J mice (**a**,**b**) were administered i.p. with leptin (5 mg kg^−1^) or PBS, and food intake was assessed for 24 h (*n*=7–8). The same mice were subsequently exposed to HFD feeding, subjected to leptin challenge tests (PBS versus Leptin 5 mg kg^−1^) after 1 day (**c**,**d**) and after 10 weeks (**e**,**f**) of HFD exposure, and monitored for body weight gain (**g**), fat mass gain (**h**) and lean mass changes (**i**). Changes in body weight (**j**), fat mass (**k**) and lean mass (**l**) were monitored in male DIO mice after AAV-induced HDAC5 overexpression in the MBH, compared with AAV–GFP control mice (*n*=10–11). Values represent means±s.e.m. Statistical analyses were done by using two-way ANOVA followed by Bonferroni *post hoc* tests (**a**–**g**,**j**), or two-tailed unpaired Student's *t*-tests (**h**,**i**,**k**,**l**). *P<0.05, **P<0.01 and ***P<0.001.

When administered leptin, chow-fed male C57BL/6J mice with AAV-mediated overexpression of GFP in the MBH had a small decrease in food intake after 8 and 12 h (Control AAV; Fig. 6a). In contrast, a substantially stronger and longer lasting leptin action was observed in mice with HDAC5 overexpression (HDAC5 AAV; Fig. 6b). To assess whether AAV-mediated overexpression of HDAC5 in the MBH prevents leptin resistance in mice fed a HFD, we exposed the same cohorts of AAV-treated mice to HFD. Consistent with previous studies that reported loss of leptin action after acute exposure to excess dietary lipids^2^,^5^,^8^,^11^,^12^,^13^, 1 day of HFD exposure was sufficient to suppress leptin (5 mg kg^−1^, i.p.) action on food intake in control AAV mice relative to saline (Fig. 6c). In contrast, mice with AAV-mediated hypothalamic overexpression of HDAC5 retained a degree of leptin sensitivity, as evidenced by a significantly reduced food intake 24 h after leptin administration compared with saline-treated HDAC5 AAV mice (Fig. 6d). Moreover, leptin sensitivity in HDAC5 AAV overexpressor mice was partially sustained even after 10 weeks of HFD exposure, compared with leptin-resistant control AAV mice (Fig. 6e,f).

Enhanced leptin sensitivity attenuated HFD-induced body weight (Fig. 6g) and fat mass gain (Fig. 6h) in AAV–HDAC5 overexpressor mice compared with AAV controls. Lean mass remained unchanged in both groups (Fig. 6i). When AAV-mediated HDAC5 overexpression was induced in the MBH of DIO mice that had consumed HFD for 6 months, there was a partial rescue of the obese phenotype, as revealed by decreased body weight (Fig. 6j), decreased fat mass (Fig. 6k) and unchanged lean mass (Fig. 6l). Overall, these findings reveal an essential role of hypothalamic HDAC5 for CNS leptin action *in vivo*, and point to HDAC5 as a critical component of body weight control in a lipid-rich environment.

## Discussion

The present experiments demonstrate for the first time that hypothalamic HDAC5 is an important component of leptin signalling and the control of food intake and body weight. Hypothalamic leptin action is of particular importance as a functional unit regulating energy metabolism^3^. Leptin-responsive circuitry in the hypothalamus originates primarily in the arcuate nucleus, an area in close proximity to the median eminence, which has a fenestrated blood–brain barrier allowing easy access to circulating nutrients and peripheral hormones. Thus, hypothalamic leptin circuitry is positioned to integrate multiple stimuli to orchestrate an adaptive response to changes in the environment. Leptin is secreted in direct proportion to adipose tissue mass and activates leptin receptors (Lep-R) residing on melanocortin circuitry, transmitting the overall metabolic status and availability of energy stores. In obese individuals, leptin-resistance blocks this feedback loop and leads to a chronically positive energy balance and excessive fat storage^8^,^11^,^12^,^13^. How environmental challenges such as HFD interfere with these complex and sensitive endogenous circuits has been the subject of intense research worldwide.

We show here that HDAC5 activity is a component of hypothalamic leptin action and essential for the normal physiological response to a HFD. When challenged with exogenous supraphysiological levels of leptin, HDAC5 KO mice are leptin resistant, while hypothalamic overexpression of HDAC5 increases leptin sensitivity. Moreover, there is reduced HFD-induced leptin resistance in mice with hypothalamic overexpression of HDAC5. Consequently, selective HDAC5 activation is a novel and promising approach to counteract leptin resistance and obesity by targeting the primary interactions between dietary challenges and the CNS control of metabolism. The finding that HDAC5 has a role in the development of leptin resistance is corroborated by a considerable series of findings revealing impaired leptin signalling following loss of HDAC5 function. Leptin receptors located on hypothalamic POMC neurons play a pivotal role in central leptin action^39^,^40^,^41^,^42^. Importantly, we also observed that mice lacking HDAC function have a consistent decrease in hypothalamic gene expression of *Pomc*, a peptide precursor that mediates leptin's anorexigenic effects. Genetic ablation of *Pomc* markedly increases food intake and body weight in mice^35^,^36^,^43^, and mutations in POMC induce severe early-onset obesity in humans^38^. Mice with *Pomc* haploinsufficiency display significant increases in food intake and body weight when exposed to a high-fat high-calorie environment^45^,^46^,^47^. Consistent with the reduction in *Pomc* gene expression, we found increased body adiposity and food intake in HFD-fed HDAC5 KO mice, and partial protection from diet-induced obesity in mice with hypothalamic overexpression of HDAC5 relative to the appropriate controls. By counting the number of POMC-expressing cells in the arcuate nucleus of HFD-fed WT and KO mice, we could conclude that changes in food intake and body weight in HDAC5 KO mice are not mediated via a loss of POMC neurons^3^,^35^,^36^,^38^,^43^, but rather via signalling cascades upstream of POMC. Our finding of a normal physiological response of HDAC5 KO mice towards pharmacological activation or inhibition of melanocortin receptor 3 and 4 signalling further suggests that leptin resistance could be mediated at the level of the leptin receptor or via downstream *Jak/Stat3* and *Pi3*k gene expression in first-order neurons. In mice with lentiviral knockdown of HDAC5 in the MBH, we observed altered expression of *Pi3k* and *Jak/Stat3*, signalling molecules mediating leptin effects on *Pomc* gene expression and food intake^27^,^28^,^29^,^33^,^44^. Consistent with this, STAT3 phosphorylation was induced in the arcuate nucleus of WT mice after leptin administration, but blunted in leptin-treated HDAC5 KO mice.

STAT3 phosphorylation is a crucial event that directly links STAT3 transcriptional activity with leptin action. Direct interactions between HDAC5 and STAT3 and a potential role for HDAC5-mediated STAT3 activation have not previously been described. Consistent with our principal hypothesis, a single report suggested STAT3-mediated recruitment of HDAC5 to the *Ptpn13* promoter^30^. We demonstrate here for the first time that HDAC5 directly associates with STAT3. Importantly, we did not use HDAC5 or STAT3 overexpression or protein tagging for co-IP experiments, thus precluding false-positive artifacts. Direct interaction of STAT3 has been reported for other HDAC family members such as Sirt1 (refs 33, 34, 36) and HDACs 1, 2 and 3 (refs 34, 37), but the overall impact of those HDAC–STAT3 interactions for the transcriptional activity of STAT3 remains controversial. Initially, STAT3 acetylation at Lys685 by the histone acetyltransferase p300 was considered critical for STAT3 dimerization and full transcriptional activity, and STAT3 deacetylation by HDACs was linked with decreased transcriptional activity^34^,^36^,^43^. However, a positive role for STAT3 acetylation stands in stark contrast to our results and several other reports that collectively and compellingly imply transcriptional repression by STAT3 acetylation. Specifically, in our hypothalamic HDAC5 KD cell model STAT3 hyperacetylation at Lysine 685 concurred with hypophosphorylation at Tyr705, decreased STAT3 binding to the *Pomc* promoter, and decreased *Pomc* expression. Other reports found that STAT3 activity decreased in renal cells after pharmacological HDAC class I inhibition^37^,^44^ and that knockdown of HDAC3 and subsequent STAT3 Lys685 hyperacetylation impaired STAT3 Tyr705 phosphorylation and decreased overall cell survival in diffuse large B-cell lymphoma^43^,^48^. Similar to our findings on STAT3, STAT1 activation and translocation was very recently linked with HDAC4-mediated deacetylation in podocytes^44^.

Exact mechanisms linking the interplay of STAT3 acetylation with phosphorylation and transcriptional activity thus appear complex, and may involve multilayered and tissue-specific processes such as differential interaction partners and differential (de)acetylation kinetics^48^,^49^. Site-directed mutagenesis of lysine residue 685 to an inactive arginine had no effect and triple mutation of lysine residues 49, 87 and 685 only slightly dampened leptin-induced STAT3 transcriptional activity^50^. The enhancement of leptin sensitivity by the transcriptional regulator Nur77 was nevertheless abolished when all three lysine residues were inactivated, potentially via differential binding of Nur77-STAT3 dimers to distant sites of the *Pomc* promoter^50^. Acetylation of Lys685 was further shown to be critical for gene expression driven by unphosphorylated STAT3^Tyr705^ (U-STAT3^Lys685^) but not by STAT3 phosphorylated on Tyr-705 (pSTAT3^Tyr705^)^51^. In hypothalamic CLU177 cells with stable knockdown of HDAC5, we observed a shift towards nuclear translocation of acetylated STAT3^Lys685^ but diminished binding of STAT3 to the *Pomc* promoter. HDAC5-mediated STAT3 deacetylation may thus control the availability of STAT3 within the nucleus, and the transactivation of genes specific for either pSTAT3^Tyr705^ or U-STAT3^Lys685^. Low intrinsic enzymatic activity of HDAC5 may further require interaction with another class I HDAC family member to catalyse STAT3 deacetylation. For hypothalamic neurons, we nevertheless conclude that direct HDAC5 action on STAT3—via reciprocal STAT3 deacetylation at Lys685 and phosphorylation at Tyr707—presents a critical step in STAT3 transcriptional activation and leptin signalling. Stat3 deacetylation—although not a classical epigenetic phenomenon—thus represents an important mechanism to convey environmental stimuli into the temporal and spatial control of gene expression.

Previous reports linked hepatic HDAC class IIa family members with the regulation of systemic glucose metabolism^17^,^49^. Mihaylova *et al*.^17^ showed that the simultaneous liver-specific knockdown of HDAC4, 5 and 7 in mouse models of diabetes type 2 could decrease gluconeogenic gene programs, increase hepatic glycogen accumulation, and improve systemic hyperglycemia. In primary murine hepatocytes, Wang *et al*.^49^ showed that the concomitant depletion of HDAC4 and HDAC5 by RNA interference could disrupt the glucagon-mediated induction of gluconeogenic gene programs. Our data from global HDAC5 KO mice do not support a specific and systemic role for HDAC5 in glucose homeostasis. The discrepant findings might be explained by redundant and compensatory action of class IIa family members 4, 7 or 9, which seem to prevent beneficial glucoregulatory effects of HDAC5 disruption in our global HDAC5 KO mouse model. Nevertheless, it should be noted that adenoviral overexpression of non-phosphorylatable constitutively nuclear HDAC5 in the liver modestly increased blood glucose in chow-fed C57Bl6 mice^17^. It remains to be determined whether global class IIa HDAC inhibitors can indeed confer beneficial changes on hepatic glucose metabolism and whether the obesogenic impact of global HDAC5 inhibition overrides any beneficial glucoregulatory effect of hepatic class IIa HDAC inhibitors.

HDAC5 is recognized as an important mediator of epigenetic changes via histone deacetylation and chromatin remodelling^25^. Histone deacetylation occurs at various sites within the histone proteins and can determine DNA packing and chromatin accessibility. Thus, silencing of HDAC5 should alter the ‘histone code' and open up chromatin, allowing for increased gene expression. HDAC overexpression should in turn decrease gene expression by condensing chromatin. Using a targeted approach, we were not able to identify upregulated expression of leptin signalling genes in HDAC5 KO mice compared with WT controls. Intriguingly, our data go beyond the classical model of epigenetic HDAC action, at least with respect to having a direct impact on leptin signalling. The possibility that modification by HDAC5 may convey additional, classically epigenetic, effects on the systemic control of metabolic homeostasis and potentially even affect metabolic programming of offspring, is a subject for future research. Ideally, such studies should involve tissue-specific HDAC5 knock-out mice to further delineate the mechanistic underpinnings of HDAC5 action.

In summary, our results identify the hypothalamic class IIa family member HDAC5 as novel regulator of metabolic homeostasis and important component of leptin signalling. Hypothalamic Hdac5 expression is regulated by nutrient availability and leptin action, revealing a fully functional feedback loop at the molecular interface that connects CNS control of metabolism with dietary challenges. Genetic and pharmacological manipulations of hypothalamic HDAC5 activity had profound effects on food intake and body-weight control in our studies and overall suggest a beneficial role for selected HDAC5 activation against leptin resistance and obesity. Mechanistically, we document for the first time a direct interaction of HDAC5 with STAT3, suggesting a STAT3 acetyl-phospho switch regulated by HDAC5. Such a switch may offer therapeutic opportunities with the potential to fine-tune leptin signalling in direct response to metabolic changes and environmental stimuli.

## Methods

### Animals

C57BL/6J mice, leptin-deficient (*Lep*^*ob*^) mice, CX3CR1–GFP^53^, GFAP–GFP^52^ and POMC–GFP mice^41^ were obtained from Janvier (Le Genest-Saint-Isle, France) or Jackson Laboratory (Bar Harbor, ME, USA). Male Wistar rats (260–290 g) were obtained from Harlan and fed chow diet througout the experiment (Indianapolis, IN, USA). HDAC5 KO mice with excision of coding exons 3 to 7 for a lacZ-neomycin resistance cassette^54^,^55^,^56^ were derived from breeding of HDAC5 heterozygous mice with pure C57BL/6J background. Excision of the Glutamine-rich N-terminal helical domain (exons 3–7, amino acids 69–156) includes all HDAC5 transcript variants currently listed as NCBI reference sequences, and suggests that HDAC5 functionality is completely ablated from our KO model. Nevertheless, we cannot fully exclude the existence of truncated HDAC5 transcript variants that lack the N-terminal domains. Mice with an age of 8 to 10 weeks were either maintained on chow (5.6% fat, LM-485, Harlan Teklad) or switched to HFD (58% kcal fat; Research Diets Inc., New Brunswick, NJ, USA) for a maximum of 16 weeks. To create DIO mice, young C57BL/6J mice were exposed to HFD for 6 months. *In vivo* experiments were performed without blinding of the investigators. All WT and KO mice used in our studies were littermates. Mice and rats were group housed on a 12:12-h light-dark cycle at 22 °C with free access to food and water, unless indicated otherwise. To assess the impact of leptin on hypothalamic HDAC5 gene expression, 6-week old leptin-deficient *(Lep*^*ob*^) mice (71.12±0.71 g) were fed chow and treated once daily subcutaneously for 6 days with either human recombinant leptin (1 mg kg^−1^; R&D Systems; *N*=6) or vehicle (PBS; *N*=6). An additional group of vehicle-treated *Lep*^*ob*^ mice (*N*=6) was food restricted to receive daily only the amount of food eaten by the leptin-treated mice (pair-fed to leptin). All studies were approved by and performed according to the guidelines of the Institutional Animal Care and Use Committee of the University of Cincinnati, USA, and the State of Bavaria, Germany.

### Body composition analysis and metabolic studies

Fat mass and lean mass were measured via Nuclear Magnetic Resonance (NMR) technology (EchoMRI, Houston, TX, USA). Energy expenditure, respiratory exchange ratio and locomotor activity were analysed by a custom-made 32-cage calorimetry system (TSE Systems, Bad Homburg, Germany)^57^. Before data collection, all mice were acclimatized to the calorimetry system for 24 h.

### Glucose tolerance and insulin tolerance tests

HDAC5 KO and WT littermates were subjected to 6 h of fasting one hour after the onset of the light phase. Subsequently, HFD-fed mice were injected intraperitoneally with 1 g glucose per kg body weight (20% wt/vol D-glucose in 0.9% wt/vol saline) for the glucose tolerance test (GTT) and 1 U insulin per kg body weight (0.1 U ml^−1^; Humolog Pen, Eli Lilly, Indianapolis, IN, USA) for the ITT. Chow-fed mice received a glucose bolus of 2 g glucose per kg body weight for the GTT. Tail blood glucose levels (mg dl^−1^) were measured with a handheld glucometer (TheraSense Freestyle) before (0 min) and at 15, 30, 60 and 120 min after injection. One male chow-fed KO mouse (Fig. 2i), two female chow-fed WT (Supplementary Fig. 3b) and one male chow-fed WT mouse (Supplementary Fig. 3c) that did not display a predefined rise in blood glucose from basal (≤50 mg dl^−1^) after a bolus injection of glucose were defined as bad injection and excluded from further analysis.

### HDAC5 knockdown studies

A set of GIPZ Lentiviral shRNA clones directed against mouse HDAC5 (Open Biosystems Huntsville, AL, USA) was assessed by western blot for optimum knockdown efficiency in the hypothalamic cell line CLU177 (mHypoA-2/12; Cellutions Biosystem Inc., Toronto, Canada). Clone V3LMM_432051 was selected for subsequent animal studies due to a knockdown efficiency of 70%. Lentiviral particles for the HDAC5 shRNA (Open Biosystems, Cat # VGM5520–200368831) and the appropriate non-silencing control (Open Biosystems, Cat # RHS4348) were injected bilaterally into the MBH of C57BL/6J mice (10 to 12-week old) (0.5 μl, 10^8^ TU ml^−1^) using a motorized stereotaxic system from Neurostar (Tubingen, Germany). Stereotaxic coordinates were −1.5 mm posterior and −0.3 mm lateral to bregma and −5.8 mm ventral from the dura. Surgeries were performed using a mixture of ketamine and xylazine (100 and 7 mg kg^−1^, respectively) as anaesthetic agents and Metamizol (50 mg kg^−1^, subcutaneous (s.c.)) followed by Meloxicam (1 mg kg^−1^, three consecutive days s.c.) for postoperative analgesia. Three days after the surgery, the single-housed mice were switched to HFD and daily food intake and body weight were recorded. A knockdown efficiency of 40 to 60% was measured by quantitative (qPCR) from hypothalamic RNA two weeks and 10 weeks after lentivirus administration, respectively.

### Intracerebroventricular injections in rats

Male Wistar rats (260–290 g) were implanted with a 22-gauge stainless-steel cannulas (Plastics One, Roanoke, VA, USA) into the third cerebral ventricle as previously described^2^,^57^. Stereotaxic coordinates were −2.2 mm posterior to bregma with a depth of −7.5 mm from the dura. For the surgery, rats were anaesthetized by using a mixture of ketamine and xylazine 86 and 12.9 mg kg^−1^, respectively). Following all surgeries, the single-housed animals received a single dose of buprenorphine (0.28 mg kg^−1^, s.c.; Buprenex; Reckitt Benckiser Healthcare, Hull, England). Correct placement of the cannula was verified by i.c.v. administration of angiotensin II (1 μg μl^−1^ of 0.9% saline). Rats that failed to drink a minimum of 5 ml of water within 30 min were removed. Food intake after i.c.v. injection of 4-Phenyl butyrate (Tocris, Ellisville, MO, USA; 4PB; 30, 100 or 300 μg dissolved in 2 μl of 0.9% saline) or vehicle was measured over a period of 4 h.

### Adenoassociated virus (AAV) infusion in mice

Custom-made AAVs with full-length murine HDAC5 complementary DNA (cDNA) under the control of a CMV promoter or an AAV with CMV–GFP as control (AAV-CMV-HDAC5 versus AAV–CMV–GFP; 2 × 10^9^ viral genome particles per ml; Sirion Biotechnology, Martinsried, Germany) were injected bilateraly into the mediobasal hypothalamus of single-housed, lean 10- to 12-week old or DIO 6-month-old C57BL/6J mice using a motorized stereotaxic system from Neurostar. Surgeries were performed using a mixture of ketamine and xylazine (100 and 7 mg kg^−1^, respectively) as anaesthetic agents and Metamizol (50 mg kg^−1^ s.c.) and Meloxicam (1 mg kg^−1^, s.c., for 3 days postoperatively) for analgaesia. Stereotaxic coordinates were −1.5 mm posterior and −0.3 mm lateral to bregma, and −5.8 mm ventral from the dura. Fluorescence microscopy of frozen hypothalamus slices confirmed GFP expression in a restricted area of the mediobasal hypothalamus (Supplementary Fig. 5a). Overexpression of HDAC5 was confirmed by western blotting from whole hypothalami; 2 weeks after AAV administration, HDAC5 protein levels were increased 1.5-fold (Supplementary Fig. 5b,c). Notably, we did not observe any compensatory increases in HDAC4 or HDAC3 protein expression in HDAC5 KO or HDAC5-AAV-treated mice, but HDAC5 KO mice displayed a restoration of Pomc expression to the levels of WT mice (Supplementary Fig. 5d,e). We corroborated HDAC5 overexpression by ipsilateral injections of HDAC5-overexpressing AAV into the MBH of POMC–GFP mice, using the contralateral, uninjected side as internal control (Supplementary Fig. 5f). Coexpression with HDAC5 was observed in 63% of all POMC–GFP neurons on the contralatral side, which was increased to 85% on the HDAC5-AAV injected ipsilateral side of the MBH (Supplementary Fig. 5g). Densitometric analyses of fluorescence intensities further revealed a 1.5-fold increase of HDAC5 protein levels on the ipsi- compared with the contralateral side (Supplementary Fig. 5h).

### Leptin sensitivity assay

Groups of HDAC5 WT and KO mice were distributed into treatment cohorts based on their starting body weight. We thereby aimed to assure an equal distribution of starting body weights at the beginning of studies, which allows for better dissection of longitudinal treatments effects on body weight or food intake. Mice were fasted 3 h before the dark. Thirty minutes before dark onset, mice were injected i.p. with 5 mg kg^−1^ leptin (R & D system, Minneapolis, MN, USA) or the PBS vehicle. At dark onset, all mice were allowed to eat, and food intake was measured throughout the dark and subsequent light period. Leptin sensitivity assays were carried out in 10- to 12-week-old HDAC5 KO and WT littermates, in control and HDAC5-overexpressor mice on chow diet, and in control and HDAC5-overexpressor mice after an acute HFD challenge of 1 day or after 10 weeks of HFD exposure.

### Chronic i.c.v. leptin and SHU9119 infusions in mice

HDAC5 KO and WT litermates were anaesthetized with a mixture of ketamine and xylazine (100 and 7 mg kg^−1^, respectively) and implanted with stainless-steel cannulas (Brain infusion kit, Alzet Durect, CA) into the lateral cerebral ventricle. The cannulas were connected via a polyvinyl tube to osmotic minipumps (model 10014D for mice; Alzet Durect, CA) that contained either vehicle (PBS), leptin (1 μg per day) or Shu 9119 (24 nmol per day, Cat. # ab141164, abcam, Cambridge, UK) and placed subcutaneously into the interscapular space, as previously described^56^. Stereotaxic coordinates were −0.7 mm posterior, −1.2 mm lateral to bregma and −2.5 mm relative to dura. Mice remained single-housed throughout the experiment. SHU9119 infusions were conducted in 8-month-old mice, leptin infusions were conducted in 10- to 12-week-old mice.

### Melanotan 2 (MT2) intraperitoneal injections in mice

Age-matched and single-housed HDAC5 WT and KO littermates (6-months old) were recorded for body weight changes after receiving daily intraperitoneal injections of saline or 1 mg kg^−1^ MTII (Cat # LKT-M1650, Enzo Life Sciences MN, USA,) for 3 days.

### Immunohistochemistry and immunofluorescence

Mice were euthanized in CO_2_ and perfused with saline, followed by a solution of 4% paraformaldehyde in 0.1 M PBS (pH 7.4) cooled to 4 °C. Brains were removed and kept in fixative at 4 °C for overnight post-fixation, then equilibrated for 48 h with 30% sucrose in 0.1 M Tris-buffered saline (TBS; pH 7.2). Brains were cut coronally in a cryostat into 30-μm sections; sections used for immunohistochemistry were collected and rinsed in 0.1 M TBS. For immunostaining, sections were sequentially pretreated with 1% NaOH with 1% H_2_O_2_ solution, 0.3% glycine and 0.3% SDS, and blocked with SUMI. Subsequently, sections were incubated overnight with primary antibody anti-pSTAT3 (rabbit polyclonal, 1:500, Cell Signaling, Danvers, MA, USA) or POMC (1:1,000, Rabbit Anti-POMC Precursor (27–52) (Porcine) Antiserum (Catalog No.:H-029-30) diluted in SUMI at 4 °C. After several TBS washes, sections were incubated with biotinylated secondary antibody diluted in SUMI (1:400, Vector Biolab, Philadelphia, PA, USA). Another round of TBS washes was followed by amplification of the signal using the avidine-biotin conjugates, and subsequent DAB-H_2_O_2_ staining for 10 min. Sections were washed with TBS, mounted on gelatine-pre-coated glass slides, and kept for air drying. Dried sections were further dehydrated by immersion in a series of alcohol (50–100%) followed by two incubation steps with 100% xylene before being mounted with a coverslip using mounting solution. Images were captured by a BZ-9000 microscope (Keyence Corporation Itasca, IL, USA). For colocalization images, hypothalamic coronal sections from POMC–GFP mice or C57Bl/6J mice were incubated with antibodies directed at HDAC5 (rabbit polyclonal, 1:1,000, Cat # 07–045, Millipore, Billerica, MA, USA) or HDAC5 and GFAP (goat polyclonal 1:1,000, Cat # SAB2500462, Sigma-Aldrich, St Louis, MO, USA), respectively. Subsequently, sections were incubated with 594 Alexa anti-rabbit or 488 Alexa anti-goat secondary antibody, and mounted on glass slides using antifade mounting media containing DAPI (Life Technologies GmbH, Darmstadt, Germany). Images were captured by either a BZ-9000 microscope or a Leica TCS SP8 Microscope and the percentage of green and red colocalized fluorescence in arcuate nucleus neurons was counted (3 slices per mouse).

### Immunocytochemistry

For immunocytochemistry, 5 × 10^4^ wildtype and knockdown CLU 177 cells were cultured in 4 well chamber slides (Lab-Tek II CC2) for 24 h, then fixed with 4% PFA and processed for immunocytochemistry. In brief, slides were washed with 3 times PBS and then incubated with blocking buffer containing 5% goat serum and 0.3% Triton X-100 for 1 h at room temperature. Subsequently, slides were incubated overnight at 4 °C with primary Ac-STAT3^K685^ antibody (Rabbit polyclonal, 1:500, Cat #2523,Cell Signaling). The slides were washed three times with PBS-T (0.01% Tween 80) and incubated with goat anti-rabbit Alexa595 fluorescence antibody in blocking buffer for 1 h at room temperature. Slides were washed three times with PBS-T and mounted with ProLong Gold Antifade Mountant with DAPI. Images were captured using a Leica TCS SP8 Microscope and analysed using ImageJ software for nuclear and cytoplasmic localization of Ac-STAT3.

### Blood chemistry

Blood was collected in EDTA-containing centrifuge tubes and centrifuged at 4 °C and 2,000*g* for 10 min to generate plasma. Plasma triglycerides, cholesterol and non-esterified fatty acids were measured by commercial enzymatic assay kits (Wako Chemicals, Neuss, Germany). Insulin and leptin were measured by ultrasensitive murine insulin and murine leptin ELISA kits (Crystal Chem, Inc., Downers Grove, IL, USA). All assays were performed according to the manufacturer's instructions.

### Hepatic triglyceride content measurement

Hepatic triglyceride content was determined by using a triglyceride assay kit (Wako Chemicals, Neuss, Germany) as described^59^.

### Cell culture, transfection and western blotting

CLU177 hypothalamic cells were cultured in DMEM supplemented with 10% fetal bovine serum and antibiotics (penicillin 100 IU ml^−1^ and streptomycin 100 μg ml^−1^) in 5% CO_2_ at 37 °C. At 70–80% confluence, cells were transfected with four different mouse GIPZ lentiviral shRNA clones (V2LMM_76750, V3LMM_432047, V3LMM_432050 and V3LMM_432051, (Open Biosystems Huntsville, AL, USA) using lipofectamine (Invitrogen, Darmstadt, Germany) for 48 h and subjected to western blotting for anti-HDAC5 (mouse monoclonal 1:2,000, Cat # sc-133106, Santacruz, CA, USA) and anti-actin (1:10,000, Cat #4970, rabbit polyclonal, Cell Signaling, Danvers, MA, USA), as previously described^58^. Subsequently, a stable cell line was made from clone V3LMM_432051 and from the appropriate non-silencing control using puromycine (10 μg ml^−1^, Sigma) treatment for 14 days. Cells were cultured in six-well plates until they reached 80–90% confluency, followed by serum starvation for 5 h and treated with and without leptin for the indicated period of time. Protein was extracted using RIPA buffer containing protease and phosphatase inhibitor cocktail (Thermo Fisher Scientific Inc., Rockford, IL USA) 1 mM phenyl-methane-sulfonyl fluoride (PMSF) and 1 mM sodium butyrate (Sigma-Aldrich, St Louis, MO, USA). Proteins were transferred on nitrocellulose membranes using a Trans Blot Turbo transfer apparatus (Biorad, Hercules, CA, USA) and stained with either anti-HDAC5 (mouse monoclonal, 1:2,000, Cat # sc-133106, Santacruz, CA, USA), or anti-POMC (goat polyclonal, 1:2,000, Cat # ab32893, abcam) or anti-Ac-STAT3^K685^ (rabbit polyclonal, 1:500, Cat #2523,), anti-p-STAT3^T705^ (rabbit polyclonal, 1:500, Cat #9145), anti-STAT3 (mouse monoclonal, 1:2,000, Cat #9139), and anti-ACTIN (1:10,000, rabbit polyclonal, Cat #4970) from Cell Signaling Technology. Detection was carried out on a LiCor Odyssey instrument (Lincoln, NE, USA) or via conventional film detection, using ECL or prime ECL (Amersham Biosciences, Piscataway, NJ, USA). Original western blots are shown as Supplementary Figs 6–9, respectively. Adenoviral delivery of HDAC5 was conducted in CLU177 cells that were first transfected with overexpresser mouse STAT3 plasmid (OriGene, Rockville, MD, USA) using lipofectamine. After 24 h of transfection, cells were incubated with recombinant replication-deficient adenovirus expressing HDAC5–GFP or control adenovirus. Cell culture media were changed after overnight incubation, and the cells were allowed another 48 h to express the transduced gene before experiments were performed.

### Primary hypothalamic neuronal cell culture

Hypothalami were extracted from Embryonic Day 14 (E14) mouse embryos. Cells were cultured separately according to the genotype of parent donors (WT or HDAC5 KO) and dissociated to single cells after digestion with trypsin, as previously described^60^,^61^. Neurons were plated on 6-well plates coated with poly-L-Lysine (Sigma-Aldrich, St Louis, MO, USA) at a density of 2,000 neurons per mm^2^, and were cultured in Neurobasal (Life technologies) supplemented with B-27 and GlutaMAX I. After 7 days in culture, glia cells were efficiently removed, and neurons started to develop synaptic processes. Cells were collected with Neurobasal without any supplements. For adenoviral deliveries of HDAC5, primary neurons at day 5 were incubated with recombinant replication-deficient adenovirus expressing HDAC5–GFP or control adenovirus. Cell culture media were changed after overnight incubation, and the neurons were allowed another 48 h to express the transduced gene before experiments were performed.

### Laser-capture microdissection microscopy

Mice were killed in CO_2_ and perfused with saline followed by a solution of 4% paraformaldehyde in 0.1 M PBS (pH 7.4, 4 °C). Brains were removed and kept overnight in fixative at 4 °C, then equilibrated for 48 h with 30% sucrose in 0.1 M TBS (pH 7.2). Brains were cut coronally in a cryostat into 40-μm sections. Section were directly mounted on polyethylene naphthalate membrane frame slides from applied biosystem and stored at −80 °C. In total, the arcuate nuclei of 16 hypothalamic slides per animal were excised and collected by using a Leica laser capture microdissection microscope. Microdissected tissues were processed for RNA isolation using the Arcturus Paradise kit (Life technologies GmbH, Darmstadt, Germany). In brief, sections were first treated with proteinase K followed by binding of RNA to the column, DNA digestion, washing and finally elution of RNA in small amounts. RNA quality and quantity were analysed by nanodrop. Equal amounts of RNA were translated to cDNA using Superscript III (Invitrogen, Darmstadt, Germany) reverse transcriptase. Gene expression was analysed using either taqman probes (Applied Biosystems, Carlsbad, CA, USA) or custom-made primers (Sigma, St Louis, MO, USA) and the respective taqman or sybr green mastermixes (Applied Biosystems). qPCRs were carried out using a ViiA 7 Real-Time PCR System (Applied Biosystems). Gene expression was evaluated using the Δ-Δ Ct method with HPRT/RPL32 as housekeeping genes.

### RNA isolation, qPCR analysis

RNA was isolated from tissues using a commercially available kit (RNeasy, Qiagen, Hilden, Germany). For qPCR analyses, equal amounts of RNA were transcribed to cDNA using Superscript III (Invitrogen, Darmstadt, Germany). Gene expression was analysed using either taqman probes (Applied Biosystems, Carlsbad, CA, USA) or custom-made primers (Sigma, St Louis, MO, USA) and the respective taqman or sybr green mastermixes (Applied Biosystems). qPCRs were carried out using a ViiA 7 Real-Time PCR System (Applied Biosystems). Gene expression was evaluated using the Δ-Δ Ct method and HPRT/RPL32 was used as housekeeping gene. Taqman probes (mouse) and primer pairs (mouse, rat) used are indicated in the Supplementary Tables 2–4, respectively.

### Immunoprecipitation

CLU177 cells were lysed with NP40 lysis buffer (25 mM Tris-HCl pH 7.4, 150 mM NaCl, 1 mM EDTA, 1% NP-40, 10 mM PMSF and 1 × protease phosphatase inhibitor cocktail). Protein was estimated by the Pierce^™^ BCA Protein Assay Kit. Cell lysates (500 μg) were diluted in lysis buffer and incubated with 4 μg of anti-HDAC5 (mouse monoclonal, Cat # sc-133106, Santacruz, CA, USA). The immunoprecipitates were incubated overnight at 4 °C with A/G magnetic beads (Invitrogen) and then washed three times with PBS containing 0.02% Tween 20. Samples were boiled in 3 × Laemmli sample buffer. Proteins were resolved on a 4–20% SDS–PAGE gel, transferred to nitrocellulose membranes (Bio-Rad), and detected with the appropriate antibodies by chemiluminescence.

### CHIP assay

Chromatin immunoprecipitation assays were performed as described before^62^,^63^. Briefly, CLU177 cells were washed with PBS, and incubated at room temperature for 45 min with 2 mM DSG and for 10 min with 1% formaldehyde before crosslinking of proteins was stopped by the addition of 0.1 M glycine. Cells were first lysed in L1 buffer (50 mM Tris pH8, 2 mM EDTA pH8, 0.1% NP-40, 10% Glycerol, protease inhibitor cocktail 10 mM sodium butyrate) to isolate nuclei. Nuclei were resuspended in L2 lysis buffer (1% SDS, 10 mM EDTA, 50 mM Tris pH 8, protease inhibitor cocktail, 10 mM sodium butyrate) and sonicated with Covaris E210 (Woburn, MA, USA) to generate fragments of genomic DNA ranging from 200 to 1,000 bp in length. Immunoprecipitations with the indicated antibodies or rabbit IgG (as control) were carried out overnight using Protein A/G mix sepharose beads (GE Healthcare, Milwaukee, WI, USA). Immunoprecipitates were washed, eluted, and then crosslinks reversed overnight at 65 °C in the presence of 1 μl RNAseA (Thermo Fisher Scientific Inc., Rockford, IL USA) and 20 μg Proteinase K. The DNA fragments were recovered using phenol:chloroform:isoamylalcohol (25:24:1) extraction followed by purification using Qiaquick PCR purification kit (Qiagen, Hilden, Germany) and subjected to RT–PCR, with primers directed against the POMC transcription start site (fwd 5′-GTTCTTCCTAACCACCAGCG-3′ and rev 5′-GTCCCTGTCGCTCTTCTCTC-3′).

### Semiquantitative measurement of HDAC5 immunostaining

Analyses of POMC HDAC5 colocalization and HDAC5 protein levels in POMC–GFP mice with ipsilateral AAV-mediated overexpression of HDAC5 were performed by using ImageJ v1.47 (http://rsbweb.nih.gov/ij/docs/guide/user-guide.pdf). Images were captured using a Leica TCS SP8 Microscope. Image intensities for HDAC5 (red fluorescence) were normalized to the non-injected contralateral side using ImageJ thresholding, and the increase in HDAC5-positive POMC neurons on the virus-injected ipsilateral side (POMC: green fluorescence; colocalization: yellow overlay) was counted manually. HDAC5 protein levels were quantified by using greyscale (8 bit) binary image conversion and thresholding followed by application of the LookUp Table function (Image>Adjust>Threshold>Apply LUT).

### Statistical analyses

All statistical analyses were performed using GraphPad Prism or SPSS. Two groups were compared by using two-tailed unpaired Student's *t*-test. Multiple comparision analyses were performed by using one- or two-way analysis of variances followed by Bonferroni *post hoc* tests. Differences in energy expenditure were assessed by analysis of covariances, with lean and fat mass as covariates. *P* values <0.05 were considered significant. All results are presented as means±s.e.m.

## Additional information

**How to cite this article:** Kabra, D. G. *et al*. Hypothalamic leptin action is mediated by histone deacetylase 5. *Nat. Commun.* 7:10782 doi: 10.1038/ncomms10782 (2016).

## Supplementary Material

Supplementary Information Supplementary Figures 1-9 and Supplementary Tables 1-4
