# Supplementary material for: Hypothalamic leptin action is mediated by histone deacetylase 5
Source: Nat Commun. 2016 Feb 29;7:10782. doi: 10.1038/ncomms10782 (PMC4773494; doi:10.1038/ncomms10782)
Supplement: Supplementary Information — Supplementary Figures 1-9 and Supplementary Tables 1-4 [file ncomms10782-s1.pdf]

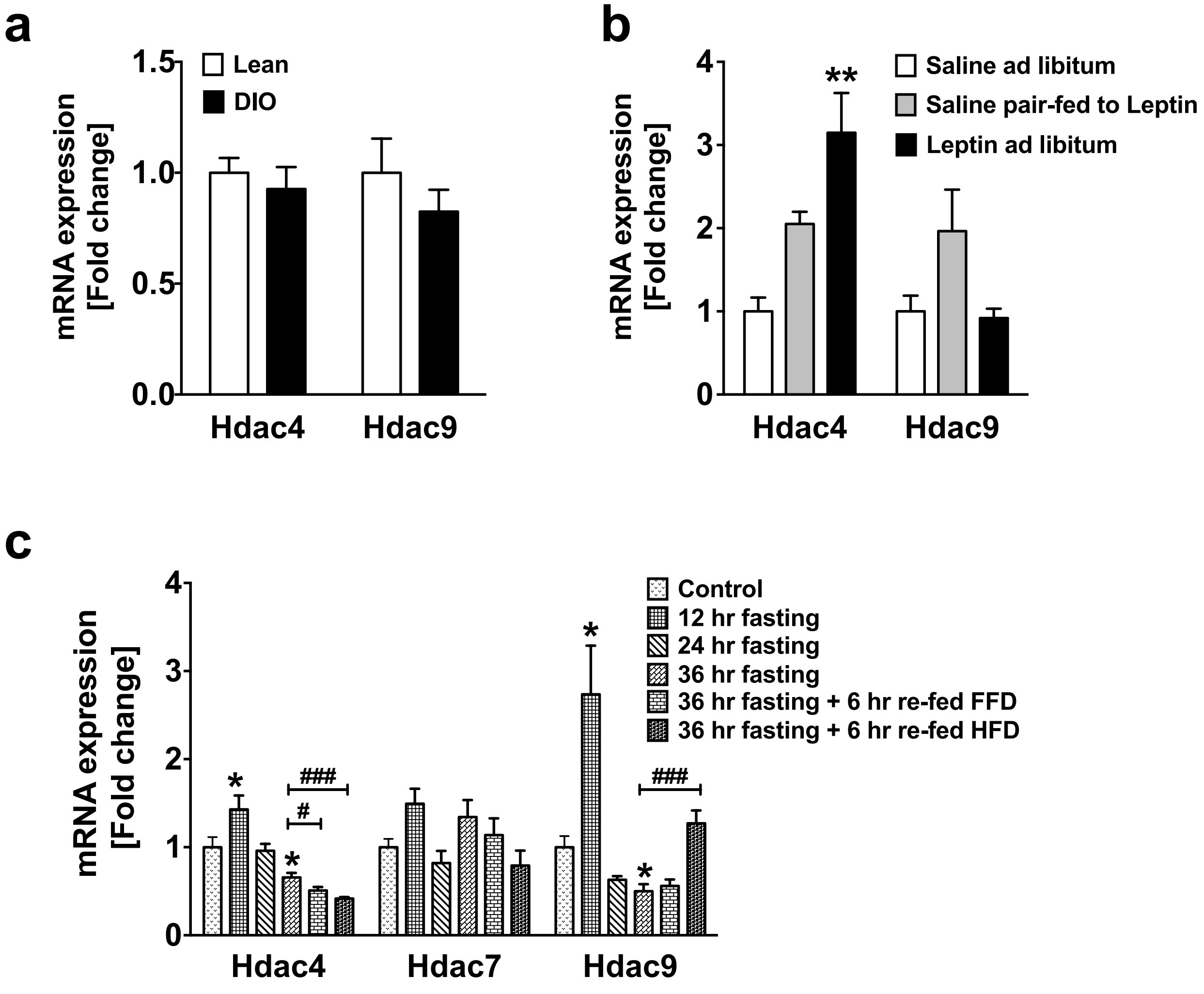

Supplementary Fig. 1: Impact of diet-induced obesity, exogenous leptin treatment and prolonged fasting and re-feeding on gene expression of HDAC class IIa family members. **(a)** Quantitative PCRs revealed unchanged hypothalamic mRNA levels of HDAC class IIa family members *Hdac4* and *9* in male C57BL/6J mice subjected to chronic chow versus high-fat diet feeding (n=7). **(b)** *Hdac4* mRNA levels were increased in leptin-deficient Lep^{ob} mice treated subcutaneously for 6 d with 1 mg/kg leptin (Leptin ad libitum), compared to ad libitum fed saline control mice (Saline ad libitum). Mice pair-fed to the lower food consumption of the Leptin ad lib group revealed a non-significant increase in

Hdac4 mRNA levels, *Hdac9* mRNA levels remained unchanged throughout all groups (n=5-6). **(c)** Hypothalamic gene expression of *Hdac4, 7* and *9* was further assessed in lean male C57BL/6J mice after prolonged fasting and re-feeding; hypothalamic *Hdac4* and *9* mRNA levels were increased slightly with short-term food deprivation but decreased after prolonged fasting. Re-feeding with fat-free diet (FFD) and high-fat diet (HFD) decreased hypothalamic *Hdac4* mRNA levels. In contrast, hypothalamic *Hdac9* levels were slightly increased with re-feeding of HFD, and *Hdac7* mRNA levels remained unaffected by changes in nutrient availability (n=6-8). Values represent means ± s.e.m. Statistical analyses were done by two-tailed unpaired Student's t tests (a), or Two-Way ANOVA followed by Bonferroni or Dunnett post-hoc tests, respectively (b,c). a,b: $^{**}p<0.01$, c: $^{*}p>0.05$ vs. Control, $^{\#}p>0.05$ 36 hr fasting vs. 36 hr fasting + 6 hr re-fed FFD, $^{\#\#\#}p<0.001$ 36 hr fasting vs. 36 hr fasting + 6 hr re-fed HFD.

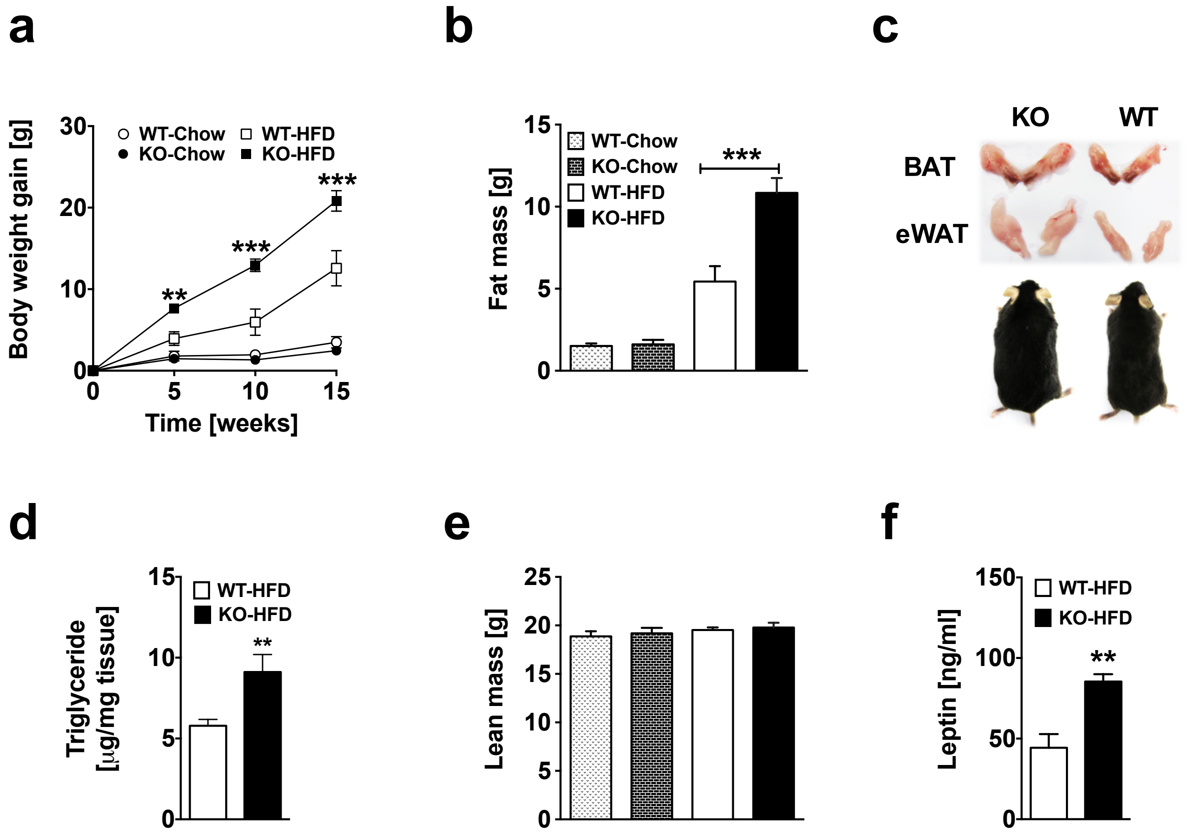

Supplementary Fig. 2: Increased adiposity in HFD-fed HDAC5 KO mice: Female HDAC5 WT and KO littermates were subjected either to chow or HFD, and evaluated for **(a)** body weight gain over a total of 15 wk; **(b)** fat mass after 8 wk of chow or HFD exposure (n=6) in female WT and HDAC5 KO mice. **(c)** Representative figures from epididymal white adipose tissue (eWAT) and brown adipose tissue (BAT) with interscapular WAT from male HDAC5 KO mice and WT littermates (n=9-10) subjected to 10 wk of HFD**. (d)** Liver triglyceride content in WT and HDAC5 KO mice (n=9-10) subjected to 10 wk of HFD **(e)** Lean mass after 8 wk of chow or HFD exposure (n=6), and **(f)** plasma leptin levels after 16 wk of HFD exposure in female HDAC5 WT and KO mice. Values represent means ± s.e.m. Statistical analyses were done by either Two-Way ANOVA followed by Bonferroni post-hoc tests (a) or or two-tailed unpaired Student's t tests (b,d,e,f). a,b: $**p<0.01$ and $***p<0.001$ WT-HFD vs. KO-HFD; d,f: $**p<0.01$.

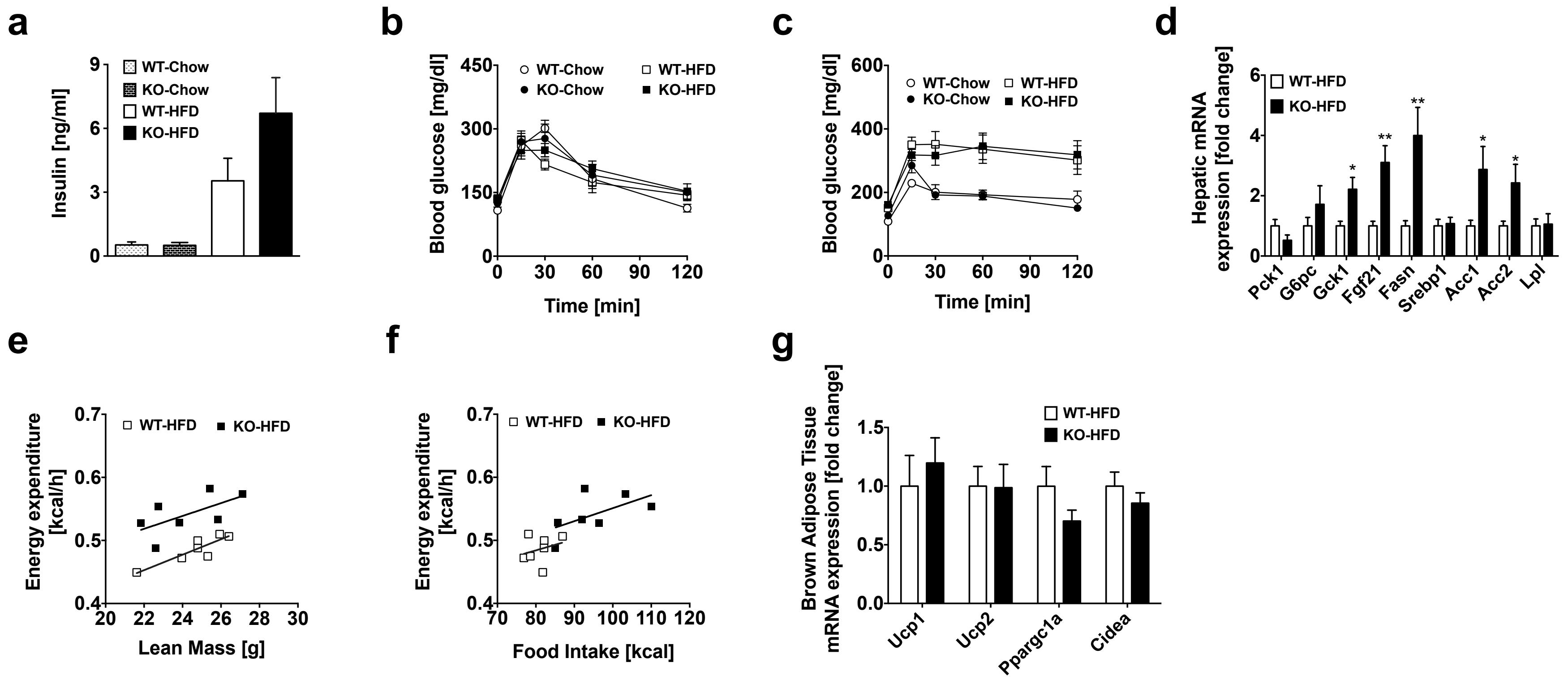

Supplementary Fig. 3: Glucose homeostasis and energy expenditure in female and male HDAC5 WT and KO mice exposed to either chow or HFD: (a) Plasma insulin levels of female HDAC5 WT and KO mice after 16 wk of chow and HFD exposure (n=5-6). **(b)** Glucose tolerance tests were carried out in female HDAC5 WT and KO mice after 15 wk of diet exposure (n=4-6). **(c)** Glucose tolerance tests after 15 wk of dietary exposure of male HDAC5 WT and KO mice to chow or HFD (n=6-8). **(d)** Hepatic gene expression of phosphoenolpyruvat carboxykinase 1 (*Pck1*), glucose-6-phosphatase (*G6pc*), glucokinase (*Gck1*), fibrioblast growth factor 21 (*Fgf21*), fatty acid synthase (*Fasn*), Sterol regulatory element-binding protein 1 (*Srebp1*), acetyl-CoA carboxylase 1 and 2 (*Acc1, Acc2*) and lipoprotein lipase (*Lpl*) was measured in male HDAC5

WT and KO mice exposed to HFD for 10 weeks (n=9-10). Energy expenditure, plotted against **(e)** lean mass or **(f)** food intake, in male HDAC5 WT and KO littermates subjected to HFD for one wk. **(g)** RNA from brown adipose tissue (BAT) of male HDAC5 WT and KO mice exposed to HFD for 10 weeks (n=7-10) was subjected to qPCR analyses of uncoupling protein 1 and 2 (*Ucp1*, *Ucp2)*, peroxisome proliferator-activated receptor gamma coactivator 1-alpha (*Ppargc1a*) and cell death-inducing dffa-like effector A (*Cidea*). Values represent means ± s.e.m. Statistical analyses were done by two-tailed unpaired Student's t-test (a, d, g), Two-Way ANOVA followed by Bonferroni post-hoc tests (b, c), or correlation analyses (e, f). *p<0.05 and **p<0.01.

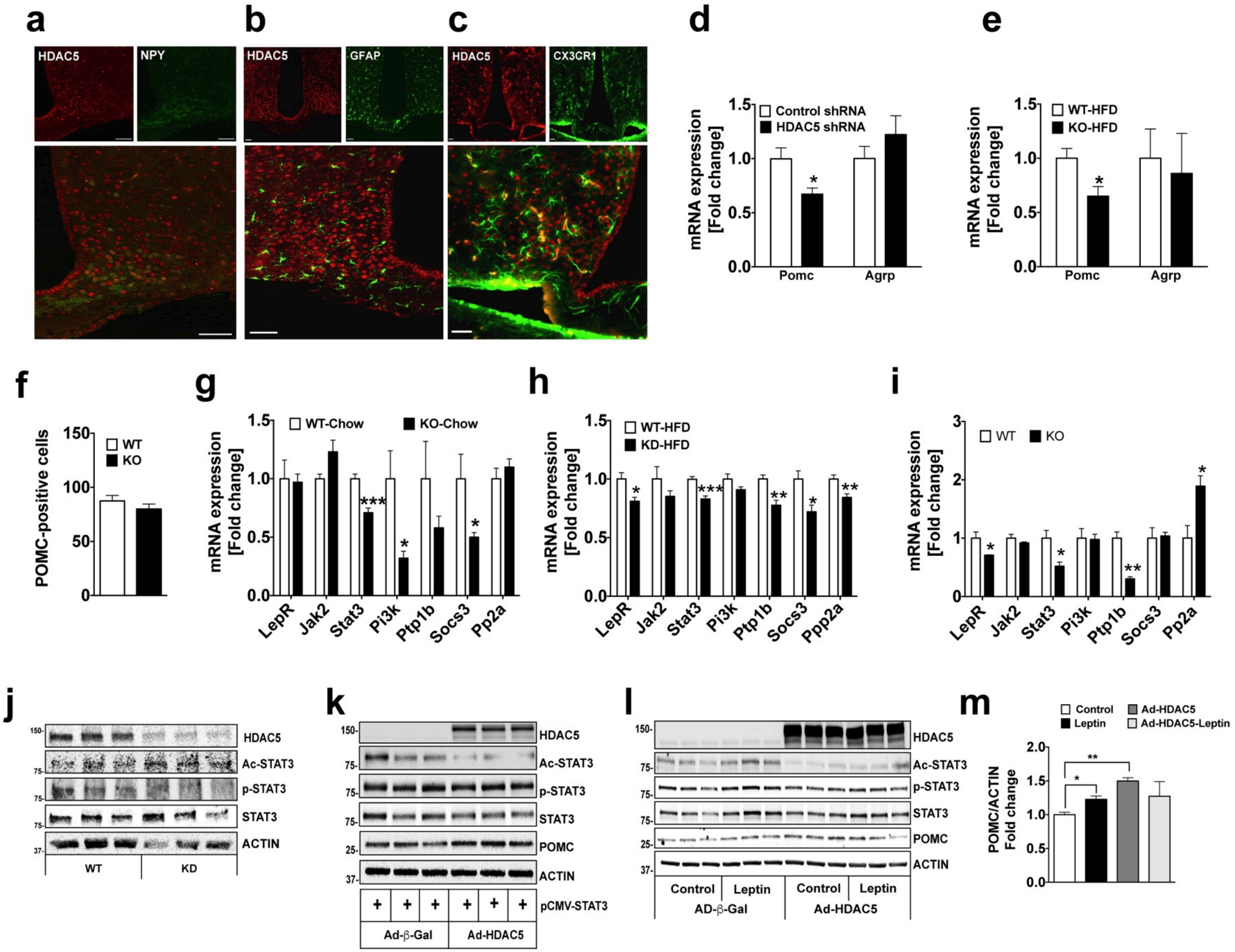

a
HDAC5
NPY
b
HDAC5
GFAP
c
HDAC5
CX3CR1
d
mRNA expression [Fold change]
Control shRNA
HDAC5 shRNA
Pomc
Agrp
e
mRNA expression [Fold change]
WT-HFD
KO-HFD
Pomc
Agrp
f
POMC-positive cells
WT
KO
g
mRNA expression [Fold change]
WT-Chow
KO-Chow
LepR
Jak2
Stat3
Pi3k
Ptp1b
Socs3
Pp2a
h
mRNA expression [Fold change]
WT-HFD
KD-HFD
LepR
Jak2
Stat3
Pi3k
Ptp1b
Socs3
Ppp2a
i
mRNA expression [Fold change]
WT
KO
LepR
Jak2
Stat3
Pi3k
Ptp1b
Socs3
Pp2a
j
HDAC5
Ac-STAT3
p-STAT3
STAT3
ACTIN
WT
KD
k
HDAC5
Ac-STAT3
p-STAT3
STAT3
POMC
ACTIN
pCMV-STAT3
Ad-β-Gal
Ad-HDAC5
l
HDAC5
Ac-STAT3
p-STAT3
STAT3
POMC
ACTIN
Control
Leptin
Control
Leptin
AD-β-Gal
Ad-HDAC5
m
Control
Leptin
Ad-HDAC5
Ad-HDAC5-Leptin
POMC/ACTIN Fold change

Supplementary Fig. 4: Hypothalamic expression and post-translational modification of HDAC5 and key leptin signaling components: Hypothalamic co-localization of HDAC5 with NPY-positive neurons **(a)**, glial fibrillary acidic protein (GFAP)-positive astrocytes **(b)** and CX3CR1-positive microglia **(c)** in coronal sections of male chow-fed NPY-GFP, C57BL/6J and microglia-GFP mice (scale bars: 50µm). Hypothalamic *Pomc* and *Agrp* mRNA levels in **(d)** C57BL/6J mice with mediobasal hypothalamic knockdown of HDAC5 compared to non-silencing controls after 10 wk of HFD exposure (n=6), or in **(e)** laser-capture-micro-dissected arcuate nucleus of male HDAC5 WT or KO mice after 16 wk of HFD exposure (n=3-4). **(f)** The number of stained cells in hypothalamic slices after immunohistochemical detection of POMC was counted in male chow-fed WT and HDAC5 KO mice (n=3). Hypothalamic mRNA expression of genes critical for leptin signaling from male HDAC5 WT and KO littermates (n=6) fed chow diet **(g,)** age (10-12 weeks), from 10-wk-HFD-fed mice (n=6) with lentiviral shRNA-mediated knockdown of HDAC5 or scramble control shRNA in the mediobasal hypothalamus **(h)**, or from primary hypothalamic neurons **(i)** obtained from HDAC5 KO and WT embryos (n=3). **(j)** Representative western blots for total HDAC, acetylation and phosphorylation of STAT3, total STAT3 and reference protein actin in WT (non silencing shNRA) and HDAC5-knockdown (KD) CLU177 cells. **(k)** Representative immunoblots from CLU177 cells transiently transfected with STAT3 and subjected to Beta-Gal control or HDAC5-GFP adenovirus (AV) treatment for 48 hr. Representative immunoblots **(l)** and densitometric analysis **(m)** from primary hypothalamic neurons subjected to transduction with beta-gal control AV or HDAC5-GFP AV followed by treatment with and without leptin (100ŋg/ml, n=3). Values represent means ± s.e.m. Statistical analyses were done by either two-tailed unpaired Student's t-test (d,e,f,g,h,i,) or One-Way ANOVA followed by Bonferroni post-hoc tests (m) . *p<0.05, **p<0.01 and ***p<0.001.

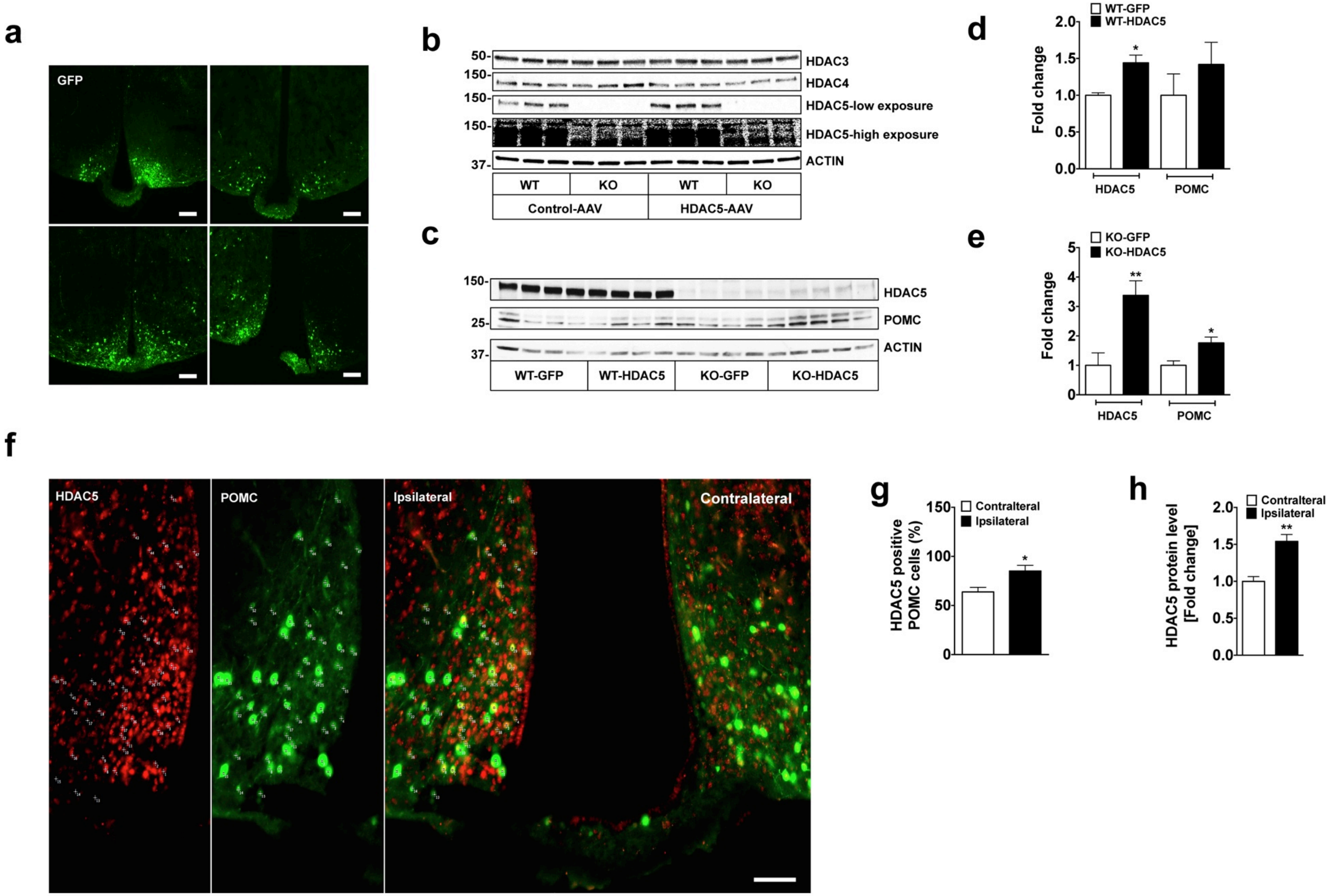
a
GFP
b
50-
150-
150-
150-
37-
HDAC3
HDAC4
HDAC5-low exposure
HDAC5-high exposure
ACTIN
WT
KO
WT
KO
Control-AAV
HDAC5-AAV
c
150-
25-
37-
HDAC5
POMC
ACTIN
WT-GFP
WT-HDAC5
KO-GFP
KO-HDAC5
d
WT-GFP
WT-HDAC5
Fold change
HDAC5
POMC
e
KO-GFP
KO-HDAC5
Fold change
HDAC5
POMC
f
HDAC5
POMC
Ipsilateral
Contralateral
g
Contralteral
Ipsilateral
HDAC5 positive POMC cells (%)
h
Contralteral
Ipsilateral
HDAC5 protein level [Fold change]

Supplementary Fig. 5: Viral overexpression of GFP or HDAC5 in the mediobasal hypothalamus of WT and HDAC5 KO mice:

(a) GFP fluorescence 2 weeks after bilateral intracranial infusion of GFP-overexpressing AAV into the mediobasal hypothalamus of C57Bl/6J mice fed chow diet (scale bar: 100µm). **(b-e)** Representative immonoblots (b: detection via Licor Odyssey; c: film detection) and densitometric analyses of ACTIN, HDAC3, HDAC4, HDAC5 and POMC in chow-fed mice with MBH-specific overexpression of GFP (Control AAV) or HDAC5. Ipsilateral infusion of HDAC5-overexpressing AAV into the MBH of chow-fed POMC-GFP mice reveals increased colocalization of HDAC5 in POMC neurons **(f,g)** and higher overall HDAC5 immunorreactivity **(h)**, compared to the uninjected contralateral side of the MBH (scale bar: 100µm). Values represent means ± s.e.m. Statistical analyses were done by two-tailed unpaired Student's t-test. *$p<0.05$, **$p<0.01$.

Fig 1d, original Western Blots

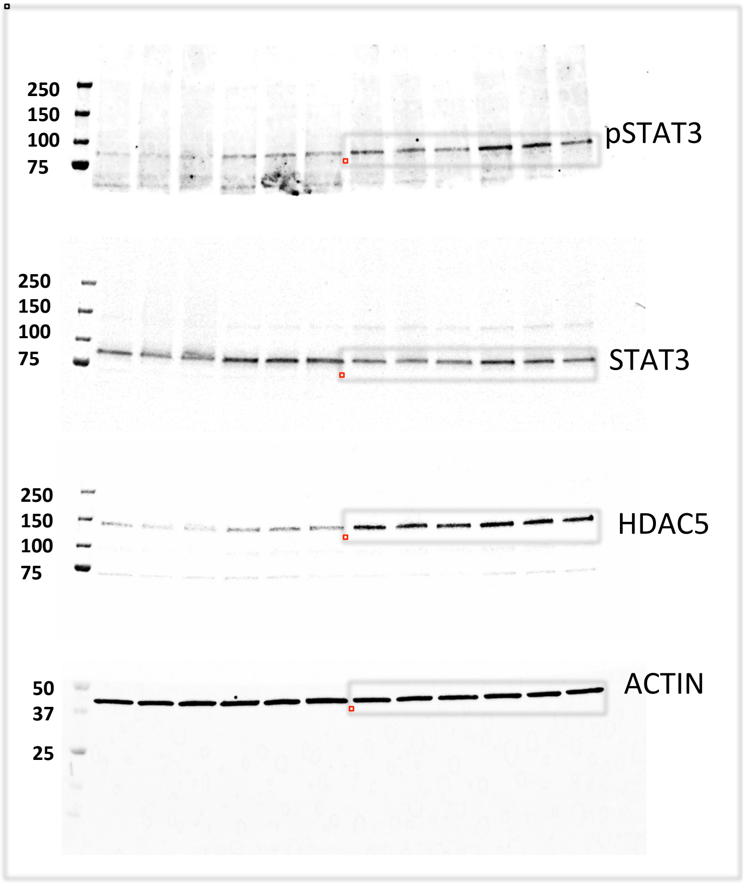

Fig 1f, original Western Blot

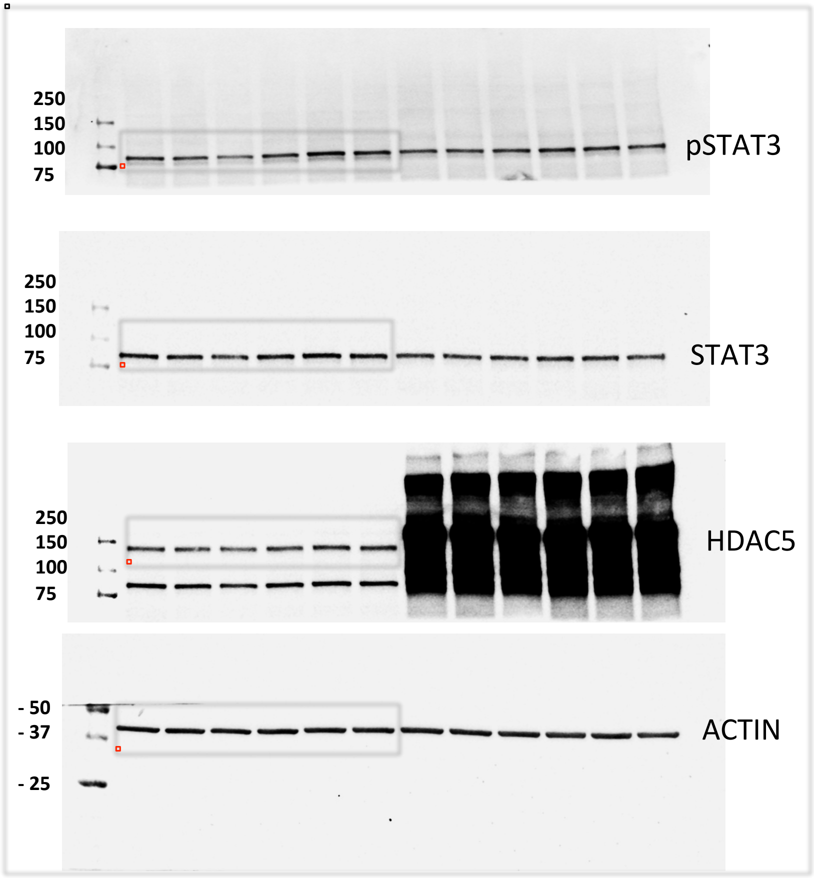

Supplementary Figure 6: Original Western Blots for figure 1, panels d and f.

Fig 4c, original Western Blot

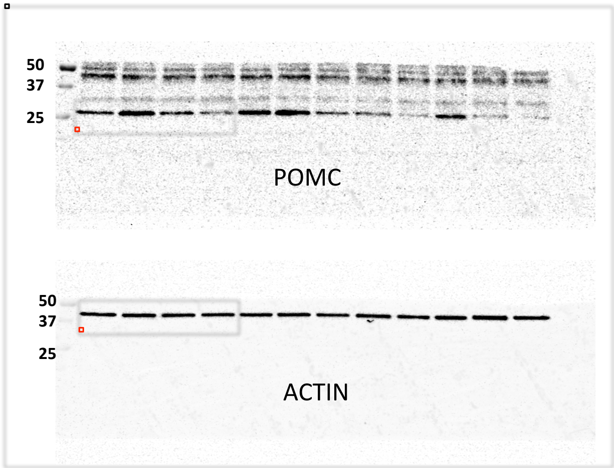

Fig. 4h, original Western Blots

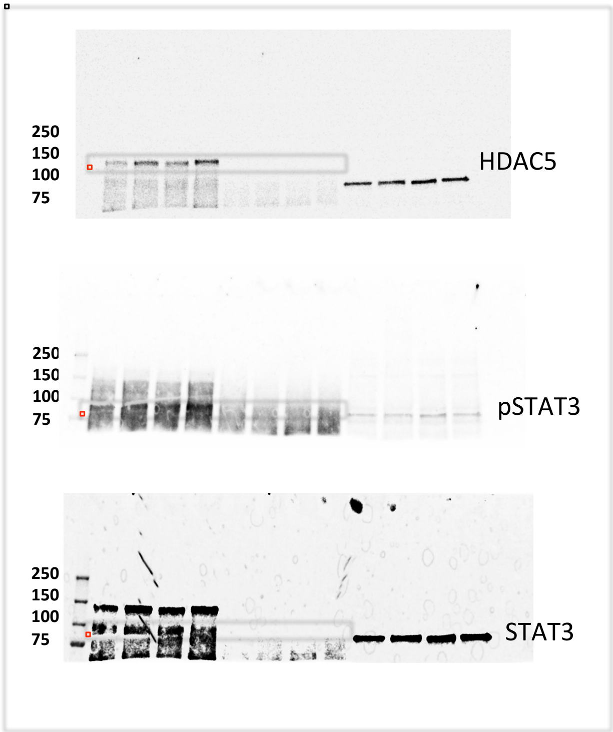

Fig 4f, original Western Blot

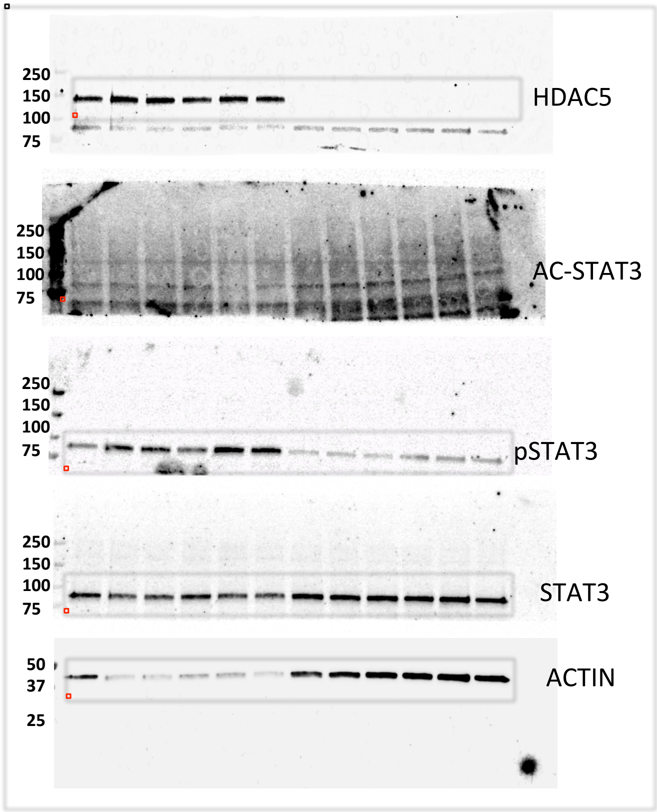

Supplementary Figure 7: Original Western Blots for figure 4, panels c, f and h.

Supplementary Figure 8

Supplementary Fig. 4j, original Western Blots

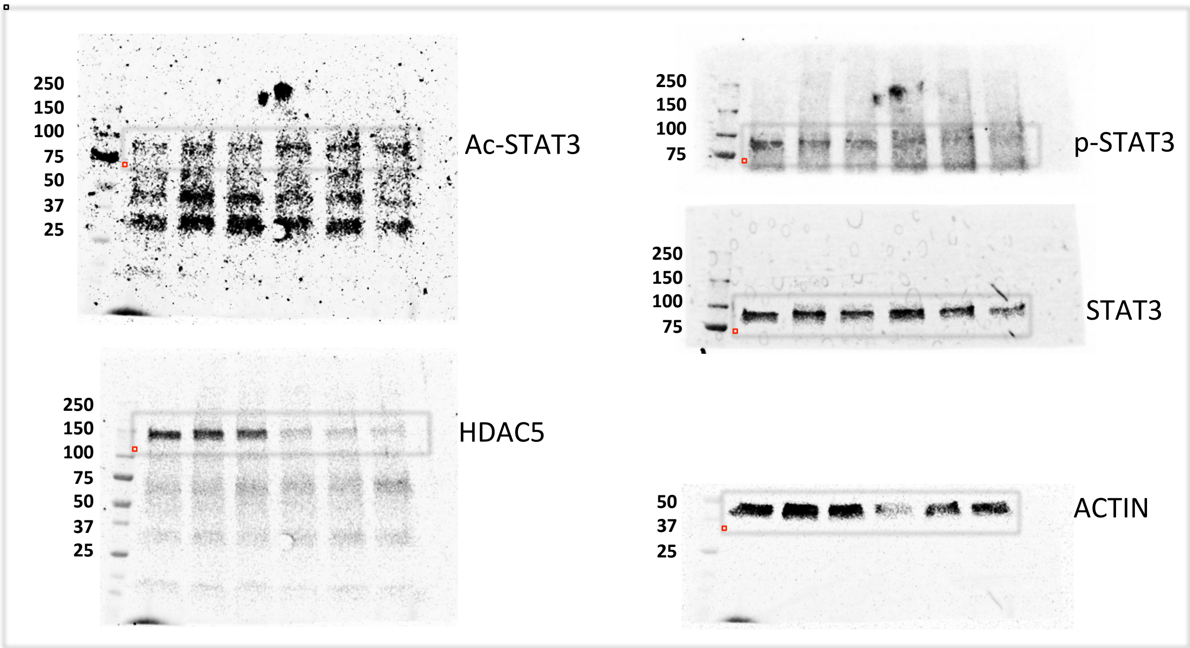

Supplementary Fig. 4k, original Western Blots

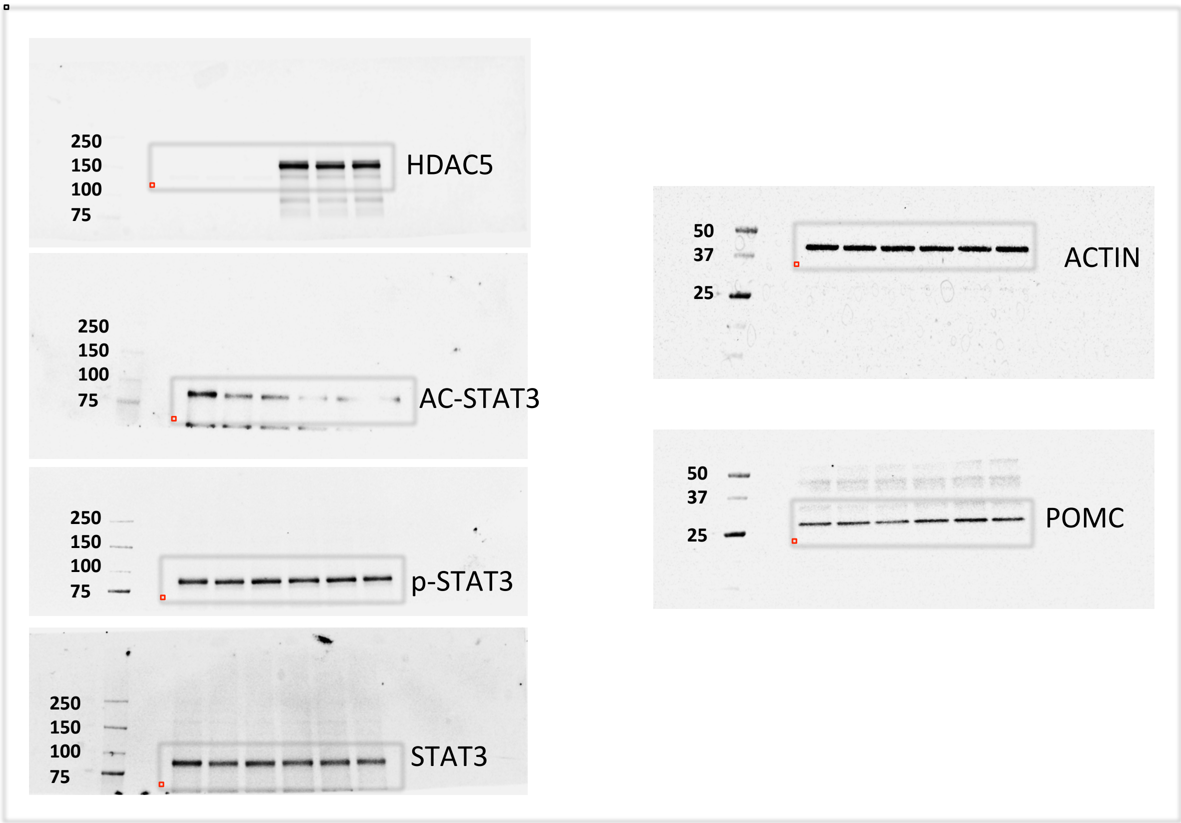

Supplementary Fig. 8, continued

Supplementary Fig. 4l, original Western Blots

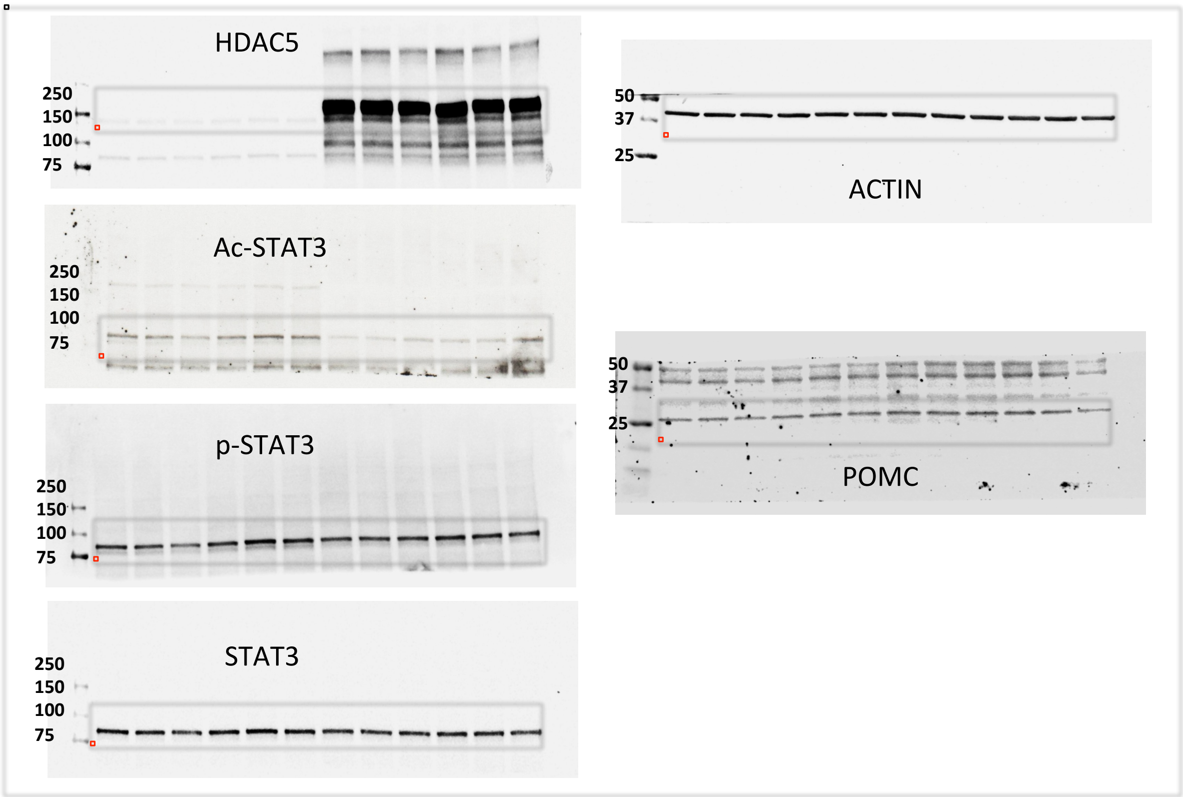

Supplementary Figure 8: Original Western Blots for supplementary figure 4, panels j, k and l.

Supplementary Fig. 5b, original Western Blots

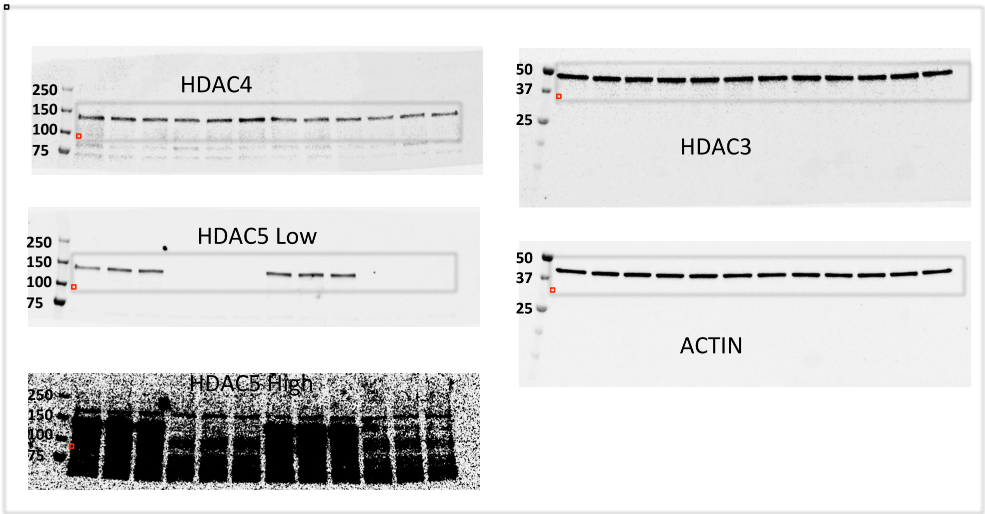

Supplementary Fig. 5c, original Western Blots

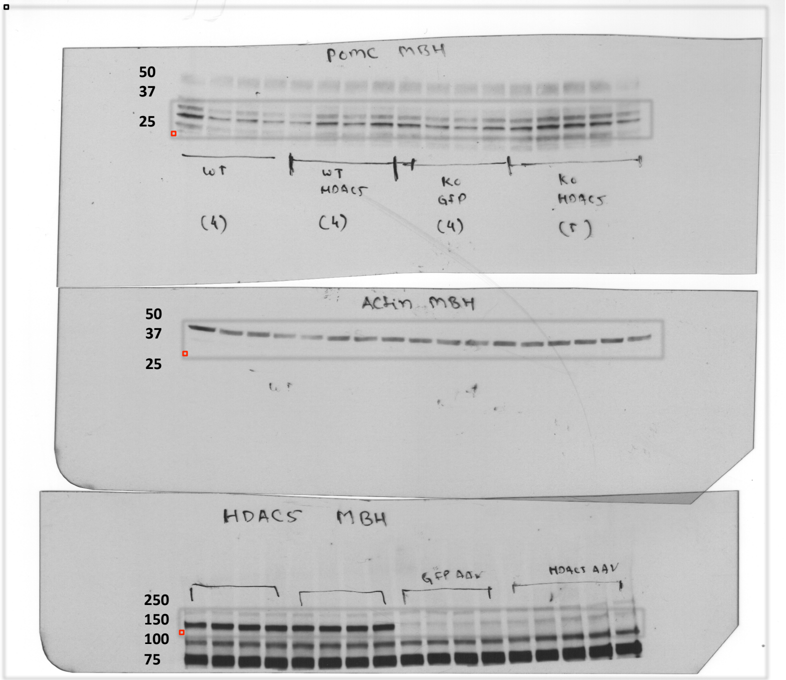

Supplemetary Fig. 9: Orignal Western Blots for supplementary figure 5, panels b and c.

Supplementary Table 1: Increased adipose tissue to body weight ratio in HDAC5 KO compared to WT: The ratio of tissue mass to total body mass was compared in male HDAC5 KO compared to WT mice at week 10 of the HFD challenge. Values represent means ± s.e.m. Statistical analyses were done using two-tailed unpaired Student's t test. *$p<0.05$, **$p<0.01$ and ***$p<0.001$

**Tissue: Body weight Ratio [g/g]**	**WT**	**KO**
Liver	0.036 ± 0.002	0.046 ±0.002 *
WAT	0.037 ±0.005	0.053 ± 0.002 *
Bat with *interscapular* fat	0.017 ± 0.002	0.036 ± 0.003 ***
BAT	0.004 ± 0.0003	0.005 ± 0.0004
Heart	0.005 ± 0.0002	0.004 ±0.0003

Supplementary Table 2: List of mouse taqman probes used for qPCR.

Gene Name	ID	Assay ID
Acetyl-CoA carboxylase 1	Acc1	Mm01304257_m1
Acetyl-CoA carboxylase 2	Acc2	Mm01204671_m1
Cell death-inducing DNA fragmentation factor, alpha subunit-like effector A	Cdiea	Mm00432554_m1
Fatty acid binding protein 4, adipocyte	Fabp4	Mm00445878_m1
Fatty acid synthase	Fasn	Mm00662319_m1
Fibroblast growth factor 21	Fgf21	Mm00840165_g1
Glucokinase	Gck1	Mm00439129_m1
Hypoxanthine phospho ribosyltransferase 1	Hprt1	Mm01545399_m1
Lipoprotein lipase	Lpl	Mm01345523_m1
Peroxisome proliferative activated receptor, gamma, coactivator 1 alpha	Pgc1a	Mm01208835_m1
Protein phosphatase 2A	Pp2a	Mm00479816_m1
Ribosomal protein L32	L32	Mm00777741_sH
Sterol regulatory element binding transcription factor 1	Srebp1	Mm00550338_m1
Suppressor of cytokine signaling 3	Socs3	Mm00545913_s1
Uncoupling protein 1	Ucp1	Mm01244861_m1
Uncoupling protein 2	Ucp2	Mm00627599_m1

Supplementary Table 3: List of mouse primer pairs used for qPCR.

Gene Name	Id	Reverse primer	Forward primer
Agouti related protein homolog	Agrp	GGCCTCAAGAAGACAACTGC	GCAAAAGGCATTGAAGAAGC
Glucose 6-phosphatase	G6Pase	GTTGAACCAGTCTCCGACC	CGACTCGCTATCTCCAAGTG
Hypoxanthine phospho- ribosyl transferase 1	Hprt1	AAGCTTGCTGGTGAAAAGGA	TTGCGCTCATCTTAGGCTTT
Janus kinase 2	Jak2	GGTGTCTGTGTCTGTGGAGA	CCCCGTTCTCCTGTCTTCTT
Leptin Receptor	LepR	CGTGGTGAAGCATCGTACTG	GGGCCATGAGAAGGTAAGGT
Phosphatidyl inositol 3-kinase	Pi3k	CACCCAAGCCCACTACTGTA	GAGTGTAATCGCCGTGCATT
Phosphoenol pyruvate carboxykinase 1	Pck1	CAGCAACTGCCCGTACTCC	CTGCATAACGGTCTGGACTTC
Proopio melanocortin	Pomc	CATTAGGCTTGGAGCAGGTC	TCTTGATGATGGCGTTCTTG
Protein-Tyrosine Phosphatase 1B	Ptp1b	CCGAGATGTCAGCCCTTTTG	CCACACCATCTCCCAGAAGT
Signal transducer and activator of transcription 3	Stat3	AATGGAAATTGCCCGGATCG	TCCTGAAGATGCTGCTCCAA

Supplementary Table 4: List of rat primer pairs used for qPCR.

Gene Name	Id	Reverse primer	Forward primer
Agouti related protein homolog	Agrp	CGACGGGTCGCAGCAAGGTA	TGGCGGAGGTGCTAGATCAG
Hypoxanthine phospho-ribosyl transferase 1	HPRT1	GTCAAGCAGTACAGCCCCAA	TGGCCACATCAACAGGACTC
Janus kinase 2	Jak2	CATGGGAATGTGTGTGCCAA	CTTGTCTCCTCCACTGCAGA
Leptin Receptor	LepR	AATCAAAATCGGCCAGCCTG	CCAGAATTCAGGCCCTCTCA
Phosphatidyl inositol 3-kinase	Pi3k	TAGTGTCCGGGAAAATGGCT	GGCATGCTCTTCGATCACAG
Proopiomelanocortin	Pomc	GAAGGTGTACCCCAATGTCG	CTTCTCGGAGGTCATCAAGC
Protein phosphatase 2A	Pp2a	CTCGTCGTACCCCAGACTAC	GCACATCTTTTGGTCCGTGT
Protein-Tyrosine Phosphatase 1B	Ptp1b	TCGACATGAAGCCAGTGACT	CCACACCATCTCCCAGAAGT
Ribosomal protein L32	L32	GGTGAAGCCCAAGATCGTCA	CAGCACTTCCAGCTCCTTGA
Signal transducer and activator of transcription 3	Stat3	TCAGTGAGAGCAGCAAGGAA	TTTCCGAATGCCTCCTCCTT
Suppressor of cytokine signaling 3	Socs3	CCTCAAGACCTTCAGCTCCA	CGACGCTCAGTGTGAAGAAG
